# A phase 2 randomised controlled trial of serelaxin to lower portal pressure in cirrhosis (STOPP)

**DOI:** 10.1186/s13063-020-4203-9

**Published:** 2020-03-12

**Authors:** Fiona J. Gifford, Philip D. J. Dunne, Graeme Weir, Hamish Ireland, Catriona Graham, Sharon Tuck, Peter C. Hayes, Jonathan A. Fallowfield

**Affiliations:** 1 Liver Unit, Royal Infirmary of Edinburgh, Edinburgh, UK; 2 Department of Radiology, Royal Infirmary of Edinburgh, Edinburgh, UK; 3 Wellcome Trust Clinical Research Facility, University of Edinburgh, Edinburgh, UK; 4 Centre for Inflammation Research, University of Edinburgh, BioQuarter, 47 Little France Crescent, Edinburgh, EH16 4TJ UK

**Keywords:** Cirrhosis, Portal hypertension, Serelaxin

## Abstract

**Background:**

In preclinical models, recombinant human relaxin-2 (serelaxin) had anti-fibrotic effects and ameliorated portal hypertension (PH). A small exploratory study in patients with cirrhosis also suggested that serelaxin could reduce portal pressure.

**Methods:**

In a phase 2, double-blind, randomised controlled study conducted in a single centre (Royal Infirmary of Edinburgh, UK), male and female adult participants with cirrhosis and clinically significant PH (CSPH; hepatic venous pressure gradient (HVPG) > 10 mmHg) were enrolled. Participants were allocated to serelaxin or placebo in a 3:1 ratio. The placebo was matched to serelaxin on appearance and administration protocol to create and maintain blinding. The primary endpoint was the change from baseline in fasting HVPG after 2 h of peripheral i.v. serelaxin infusion (80 μg/kg/day for 60 min followed by 30 μg/kg/day for at least 60 min). Secondary endpoints included the change from baseline in hepatic blood flow and systemic haemodynamics (cardiac index, systemic vascular resistance index and aortic pulse wave velocity). Short-term safety and tolerability of serelaxin were assessed.

**Results:**

A total of 17 participants were screened, 15 were randomised and 11 completed the study (*n* = 9 serelaxin, *n* = 2 placebo). Reasons for withdrawal were baseline HVPG < 10 mmHg (*n* = 2) and technical failure (*n* = 2). The trial ended early due to manufacturer discontinuation of the study drug. The median age was 56 (range 43–69) years and 73% of participants were male. Alcohol was the commonest cirrhosis aetiology (*n* = 10). Participants had a median Model for End-Stage Liver Disease score of 10 (range 6–14). The mean baseline HVPG was 16.3 (range 10.3–21.7) mmHg. Individual responses were variable, but overall there was no statistically significant change in HVPG after 2 h of i.v. serelaxin (arithmetic mean of difference ± SD was 0.4 ± 3.5 mmHg (95% CI –2.3, 3.1; *p* = 0.76)). There were also no substantial changes from baseline in hepatic or systemic haemodynamics. We recorded 12 adverse events in 7 participants treated with serelaxin; none were significant, and most were unrelated to the investigational medicinal product. There were no serious adverse events.

**Conclusion:**

In a small randomised, phase 2, proof-of-concept study in patients with cirrhosis and CSPH, serelaxin infusion was safe and well-tolerated but had a neutral effect on HVPG.

**Trial registration:**

ClinicalTrials.gov, NCT02669875. Registered on 1 February 2016.

## Background

Standardised mortality rates for liver disease in the UK have increased 400% since 1970, and in people younger than age 65 years have increased by almost 500% [1]. In patients with cirrhosis of the liver, portal hypertension (PH) is the main cause of death and of liver transplantation. In Europe alone, it is estimated that 29 million patients suffer from chronic liver disease, and that 170,000 die each year from complications of cirrhosis, a number exceeding the mortality due to breast cancer [2]. Patients with hepatic venous pressure gradient (HVPG) ≥ 10 mmHg (clinically significant PH, CSPH) are at increased risk of hepatic decompensation [3] and of hepatocellular carcinoma [4]. Variceal bleeding occurs when the HVPG is > 12 mmHg. A reduction in the HVPG to < 12 mmHg or by > 20% from baseline is reported to improve clinical outcomes and represent targets for haemodynamic response in interventional studies [5]. Despite a significant improvement in outcomes over the past 30 years, the average 6-week mortality for the first episode of variceal bleeding in most studies is reported to be up to 20% [6].

Terlipressin, a synthetic analogue of vasopressin, has an immediate systemic vasoconstrictor action followed by portal haemodynamic effects due to slow conversion to vasopressin. It is the only pharmacological agent used in acute variceal bleeding that has been shown to reduce mortality in placebo-controlled trials [6]. Terlipressin decreases failure of initial haemostasis by 34%, decreases mortality by 34% and is considered a first-line treatment for bleeding oesophageal varices, when available. However, off-target effects include peripheral and coronary ischaemia, and adverse events (AEs) occur in 10–20% of patients [7]. Terlipressin is not licensed in the USA, where octreotide (a somatostatin analogue) is most commonly used. Octreotide is also thought to act as a mesenteric arterial vasoconstrictor, but in an acute haemodynamic study, octreotide was found to only transiently reduce HVPG and portal venous flow [8]. Nevertheless, octreotide has recently been shown to be as effective as terlipressin in the control of acute variceal bleeding [9].

We have previously shown that serelaxin, a recombinant form of the human peptide hormone relaxin-2, had anti-fibrotic and portal hypotensive effects in cirrhotic rats [10]. Moreover, serelaxin reduced the portal pressure by decreasing intrahepatic vascular resistance (IHVR) through augmentation of nitric oxide (NO) bioavailability and signalling, thus maintaining or enhancing hepatic blood flow. In a recent small, exploratory, open-label, phase 2 study [11], Part B showed that serelaxin induced a rapid and potentially clinically significant reduction in portal pressure in patients with cirrhosis, PH and a transjugular intrahepatic portosystemic shunt (TIPSS). Following at least 120 min of serelaxin infusion there was a 31.3% (95% CI –66.5, 71.6) reduction in the portal pressure gradient (PPG) compared to baseline. During the infusion there was a progressive reduction in the portal vein pressure (PVP), reaching a decrease of 25.2% (95% CI –12.7, 50.3) from baseline at the 120-min time point. The reduction in PVP started at 30 min and continued through to the 135-min time point. With serelaxin infusion, there were no newly occurring liver enzyme abnormalities, no clinically significant changes in blood pressure and no discontinuations due to AEs. Indeed, in a separate study, the pharmacokinetic and safety profiles of serelaxin were not affected in patients with mild, moderate or severe hepatic impairment [12].

The objective of this double-blind, randomised placebo-controlled study was to evaluate, for the first time, the safety and efficacy of serelaxin in reducing the portal pressure, as determined by the HVPG in patients with cirrhosis and CSPH.

## Methods

### Study participants

We enrolled male or female adult participants over the age of 18 years with cirrhosis and PH. Full eligibility criteria are listed in Additional file 1. The main inclusion criteria included: clinically diagnosed or biopsy-proven liver cirrhosis of any aetiology; evidence of PH either on imaging or on previous endoscopy; and suspected HVPG ≥ 10 mmHg at baseline (if the baseline HVPG was subsequently found to be < 10 mmHg, the participant was withdrawn from the study). The main exclusion criteria included: pregnancy or breast-feeding; women of child-bearing potential not using highly effective methods of contraception; severe liver failure; history of variceal bleed within the previous month; hepatocellular carcinoma or history of malignancy of any organ system (other than localised basal cell carcinoma of the skin); portal vein thrombosis; previous surgical shunt or TIPSS; current use of beta-blockers or nitrates, or any other drug therapy known to have an influence on portal pressure (diuretics were permitted provided patients had been on a stable dose for at least 30 days); history of active/recent drug or alcohol abuse; sitting systolic blood pressure < 110 mmHg at screening visit or within 10 min prior to starting study drug infusion; significant arrhythmias, including prolonged QT interval; documented hypersensitivity to i.v. contrast agents and/or iodine; severe renal impairment; significant structural heart disease (including cardiomyopathy, valvular disease); major neurologic events, including cerebrovascular events, within the previous month; clinical evidence of acute coronary syndrome currently or within the previous month; and pacemaker, cardiac resynchronisation device or implantable cardioverter-defibrillator in situ.

### Data collection

This was a single-site study, undertaken at the Royal Infirmary of Edinburgh, Edinburgh, UK between 19 October 2017 and 15 August 2018.

Participants attended the RIE Clinical Research Facility for screening (visit 1) consisting of a physical examination, blood tests (full blood count, coagulation and biochemistry), electrocardiogram (ECG), blood pressure measurement and written informed consent. Randomisation was performed once it was known that the participant had passed screening, prior to the study visit.

On the study day (visit 2; ≤ 7 days after the screening visit), eligible participants attended for baseline haemodynamic measurements, following an overnight fast and the avoidance of caffeine for > 8 h. After baseline evaluation and confirmation of HVPG ≥ 10 mmHg, participants received (in a double-blind fashion) either serelaxin or placebo. The haemodynamic measurements were repeated at specified time points. A peripheral blood sample was taken at baseline and after 2 h, processed and stored for potential future analysis. After the post-treatment assessments, participants were observed for a recovery period of 4 h which included repeat physical examination, blood pressure, ECG measurement and routine laboratory blood tests. There was no follow-up visit. Participants were contacted by a member of the research team via telephone at 24 h and again at 4 weeks after the study visit to collect information about potential AEs and concomitant medications.

Study data were collected and managed using REDCap electronic data capture tools hosted at The University of Edinburgh. REDCap [13] (Research Electronic Data Capture) is a secure, web-based application designed to support data capture for research studies, providing: an intuitive interface for validated data entry; audit trails for tracking data manipulation and export procedures; automated export procedures for seamless data downloads to common statistical packages; and procedures for importing data from external sources.

### Study design, randomisation and allocation concealment

This study was a phase 2, double-blind, randomised controlled trial to investigate the effects of serelaxin on PH in patients with cirrhosis. Randomisation was carried out by an independent third party (Edinburgh Clinical Trials Unit) after it was confirmed that the participant had passed screening, prior to the study visit (visit 2). Random sequences of block sizes were generated by computer to achieve a 3:1 allocation ratio between serelaxin and placebo; there was no stratification to this allocation. Randomisation produced a four-digit integer matching a bottle number held by the pharmacy. The Investigators had no way of linking the drug allocation to the four-digit number. The person generating the randomisation list and allocation concealment was not involved in the later implementation of the sequence. The original randomisation list was held in a secure folder with restricted access to the Edinburgh Clinical Trials Unit Data Management team. The placebo was matched to serelaxin on appearance and administration protocol to create and maintain blinding.

### Sample size

The primary efficacy endpoint was the decrease in the fasting HVPG between baseline and 2 h post serelaxin treatment, targeting for a 20% reduction. The sample size calculation was based on a previous study in Edinburgh evaluating carvedilol [14] and the data from the previous Novartis-sponsored serelaxin phase 2 study (ClinicalTrials.gov identifier: NCT01640964) [11]. Assuming a mean baseline HVPG of 16.37 (SD = 2.14) mmHg and a post-baseline HVPG of 13.1 (SD = 3.91) mmHg (20% decrease), the change from baseline in the HVPG was estimated to be 3.3 (SD = 4) mmHg. A sample size of 14 participants in the serelaxin group would provide 80% power to detect at least a 20% decrease from baseline in HVPG using a two-sided paired *t* test with an α level of 0.05. A small number of placebo-treated patients were included in order to preserve double-blindness, not as a comparison group. Therefore, it was proposed that a total of 20 patients (15 serelaxin and 5 placebo) would be randomised in a 3:1 ratio.

### Intervention

Recombinant human relaxin-2 (serelaxin; Novartis Pharmaceuticals, UK) or placebo (20 mM sodium acetate buffer solution at pH 5.0; Novartis Pharmaceuticals, UK) were administered via peripheral i.v. infusion at two different infusion rates: 80 μg/kg/day for 60 min followed by 30 μg/kg/day for at least 60 min (until completion of the final HVPG/ICG measurements). This was achieved by a single infusion bag, prepared by the clinical trials pharmacist, with a uniform change in the administration rate.

### Study endpoints

The primary endpoint was the change from baseline in the fasting HVPG after 2 h of serelaxin infusion. The HVPG was measured as previously described [14]. The procedure was performed after overnight fast and at the same time of day due to circadian variation in HVPG measurements. Prior to catheter insertion, participants were offered low-dose (≤ 0.02 mg/kg) midazolam to reduce any anxiety. A 7-F venous introducer was inserted into the right femoral vein using the Seldinger technique under ultrasound guidance. A balloon-tipped catheter was then advanced into a hepatic vein using fluoroscopy. The free hepatic venous pressure (FHVP) was measured with the balloon deflated and floating freely in the hepatic vein close to its junction with the inferior vena cava (IVC). The wedged hepatic venous pressure (WHVP) was measured with the balloon inflated until the branch of hepatic vein was completely occluded. The HVPG was obtained by subtracting the FHVP from the WHVP. All measurements were performed in triplicate and permanent tracings were printed, stored and read blindly at the end of the study prior to the opening of the randomisation codes. The HVPG was measured at baseline, and then repeated after 60 and 120 min of the IMP (either serelaxin or placebo). Baseline HVPG ≥ 10 mmHg confirmed the presence of CSPH. If HVPG < 10 mmHg was obtained, the study participant was withdrawn. The IVC pressure (IVCP) was measured at baseline and after the final HVPG measurement.

Secondary endpoints included: the change from baseline in the fasting HVPG after 1 h of serelaxin infusion; the change from baseline in the fasting hepatic blood flow (HBF) after 2 h of serelaxin infusion (measured from the concentration of indocyanine green (ICG) in the hepatic venous blood vs peripheral venous blood using the Fick Principle); the change from baseline in the IVCP after 2 h of serelaxin infusion; the change from baseline in the cardiac index (CI) after 2 h of serelaxin infusion; the change from baseline in the systemic vascular resistance index (SVRI) after 2 h of serelaxin infusion; the change from baseline in the aortic pulse wave velocity after 2 h of serelaxin infusion; safety and tolerability of serelaxin infusion (as assessed throughout the study by monitoring AEs, clinical laboratory blood tests, heart rate, blood pressure and ECG); and the change from baseline in exploratory blood biomarker measurements after 2 h of serelaxin infusion (if a demonstrable effect on the HVPG was observed).

The total HBF was calculated using the ICG constant infusion method [15, 16] and derived from measurements of ICG clearance and extraction. Baseline serum samples were taken prior to each ICG infusion. Thereafter, 10 mg of ICG (10 ml) was given as a slow i.v. bolus via a peripheral cannula, followed by an infusion of 0.2 mg/min (0.2 ml/min or 12 ml/h) by accurate infusion pump (Alaris Asena, Becton Dickinson, USA). After an equilibrium period of at least 40 min, samples were taken simultaneously from the right hepatic vein (via the catheter tip) and the femoral vein (via the side port of the introducer). Paired samples (hepatic and femoral) were taken in triplicate, 2 min apart, in order to confirm equilibrium. The HBF was measured in this way both at baseline and after 120 min of IMP infusion.

A Cardioscreen 1000 (Medis, Germany) was used for non-invasive measurement of cardiac output (CO (L/min) = heart rate × stroke volume / 1000), CI (L/min/m^2^) and SVRI (dyne·s·cm^5^·m^2^) by the bio-impedence technique before and after 120 min of IMP infusion. Similarly, arterial function was measured using an Arteriograph device (TensioMed, Hungary). Arterial stiffness and central haemodynamics were assessed by the application of an inflatable cuff to the upper arm. The aortic pulse wave velocity (APWV, m/s) was calculated as the distance the pulse wave travels in the aorta (as measured from the suprasternal notch to pubic bone) divided by the measured transit time.

Participants were monitored for 4 h after IMP discontinuation and removal of the catheter and introducer from the femoral vein. Vital signs were recorded every 30 min throughout the infusion and recovery periods, with regular ECG monitoring. Adverse events (AEs) were collected during visit 2 and by follow-up telephone calls at 24 h and 4 weeks. The severity, expectedness and causality of AEs in relation to the study medication were noted by the study team. The Investigators and the co-sponsors had the right at any time to terminate the study for clinical or administrative reasons.

### Statistical analysis

Analysis was per protocol. The number of participants who were withdrawn by the investigator during the course of the study is presented broken down by treatment allocation and presented with reasons for withdrawal where available. Missing data as a result of patients not having the post-baseline measurement were not imputed. Patients with missing post-baseline data were excluded from the analysis at that time point.

Summary statistics (*n*, mean, SD, median, minimum, maximum, Q1 and Q3) were generated over time for the baseline, post-baseline and change from baseline measurements for the primary endpoint in the serelaxin and placebo group. The geometric mean was calculated for the baseline value, the post-baseline values and the ratio to the baseline values. Confidence intervals were calculated for both the arithmetic and geometric means. Paired *t* tests were used to test the mean change from baseline measurements. The secondary endpoints were subjected to the same analysis as the HVPG. For the placebo control group, the change from baseline to 2 h was analysed in the same way as the primary outcome, although, as this was not powered for, no direct statistical comparison was made between serelaxin and placebo.

There were no planned interim analyses for safety or efficacy. All participants were analysed in the group to which they were originally assigned irrespective of the treatment received, with the exception of AEs which are presented according to allocated treatment and also treatment received. For all analyses, unless otherwise specified, statistical significance was taken to be *p* < 0.05.

## Results

### Participant flow

**Fig. 1:**
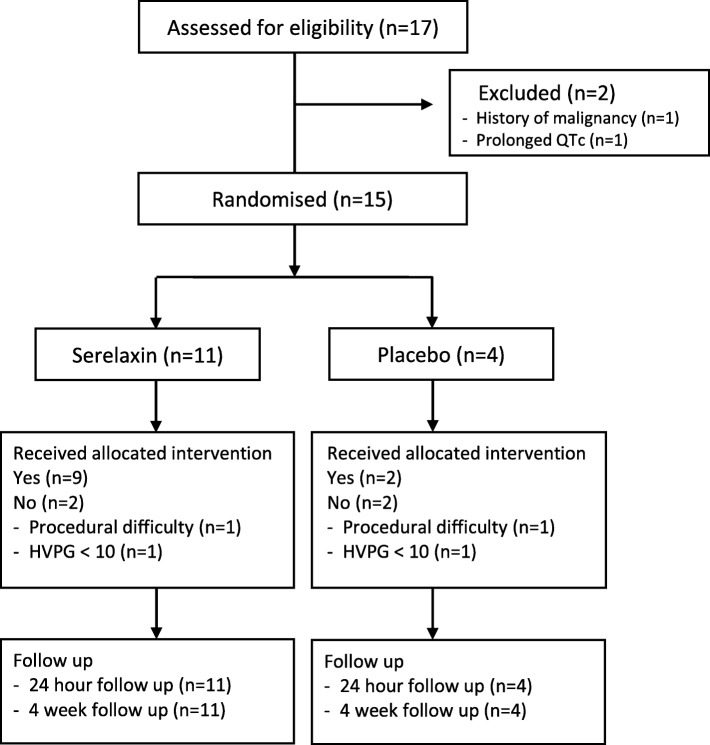
Consort flow diagram. HVPG hepatic venous pressure gradient, QTc corrected QT interval

Participant disposition is shown in the CONSORT diagram (Fig. 1). A total of 17 participants were recruited. Of these, two participants had a screening failure and did not proceed to randomisation. Fifteen patients were randomised and 11 completed the trial (*n* = 9 serelaxin, *n* = 2 placebo). Four participants were withdrawn by the investigators following randomisation, did not receive any study drug and are not included in any primary or secondary analyses. Reasons for withdrawal were baseline HVPG < 10 mmHg (*n* = 2) and HVPG technical failure (*n* = 2). The trial ended early due to manufacturer discontinuation of the study drug.

### Baseline participant data

Participant characteristics are summarised in Table 1. The median age of participants was 56 (range 43–69) years and 73% were male. Cirrhosis aetiologies were alcohol-related liver disease (*n* = 10), non-alcoholic fatty liver disease (*n* = 2), chronic hepatitis C (*n* = 2) and chronic hepatitis B (*n* = 1). Participants were Child–Pugh class A (60%) and class B (40%) with a median MELD score of 10 (range 6–14). The mean baseline HVPG was 16.3 (range 10.3–21.7) mmHg.

**Table 1:** Summary of participant characteristics

	All participants (*n* = 15)	Serelaxin (*n* = 11)	Placebo (*n* = 4)
Age (years)	56 (43–69)	56 (43–69)	59 (54–63)
Gender (% male)	11 (73%)	8 (73%)	3 (75%)
Ethnicity (% Caucasian)	15 (100%)	11 (100%)	4 (100%)
Aetiology of cirrhosis			
Alcohol alone	10 (67%)	8 (73%)	2 (50%)
NAFLD	2 (13%)	1 (9%)	1 (25%)
HCV alone	1 (7%)	1 (9%)	0
HCV + HBV	1 (7%)	0	1 (25%)
Cryptogenic	1 (7%)	1 (9%)	0
Child–Pugh class A	9 (60%)	6 (54%)	3 (75%)
Child–Pugh class B	6 (40%)	5 (45%)	1 (25%)
Child–Pugh class C	0	0	0
Current/previous liver-related complications			
Ascites	8 (53%)	6 (55%)	2 (50%)
Spontaneous bacterial peritonitis	0	0	0
Hepatic encephalopathy	4 (27%)	3 (27%)	1 (25%)
Variceal bleeding	5 (33%)	5 (45%)	0
BMI (kg/m^2^)	27.6 (19.8–36.6)	28.0 (24.1–36.6)	26.5 (19.8–31.8)
Systolic BP (mmHg)	145 (112–173)	155 (126–173)	134 (112–149)
Heart rate (bpm)	71 (46–97)	73 (46–97)	69 (66–71)
MELD score	10 (6–14)	11 (8–14)	7.5 (6–11)
Total bilirubin (μmol/L)	24 (6–44)	28 (7–44)	12 (6–17)
INR	1.2 (1.0–1.6)	1.3 (1.1–1.6)	1.2 (1–1.5)
Albumin (g/dL)	34 (23–40)	34 (23–39)	36 (30–40)
Platelet count (× 10^9^/L)	71 (26–331)	71 (26–182)	104 (55–331)
AST (U/L)	43 (22–122)	46 (25–122)	28 (22–32)
ALT (U/L)	37 (10–123)	37 (12–123)	26 (10–39)
Ongoing alcohol use			
Yes	4 (27%)	3 (27%)	1 (25%)

Data presented as median (range) or *n* (%)

*ALT* alanine aminotransferase, *AST* aspartate aminotransferase, *BMI* body mass index, *BP* blood pressure, *bpm* beats per minute, *HBV* hepatitis B virus, *HCV* hepatitis C virus, *INR* international normalised ratio, *MELD* model for end-stage liver disease, *NAFLD* non-alcoholic fatty liver disease

### Primary endpoint

**Fig. 2:**
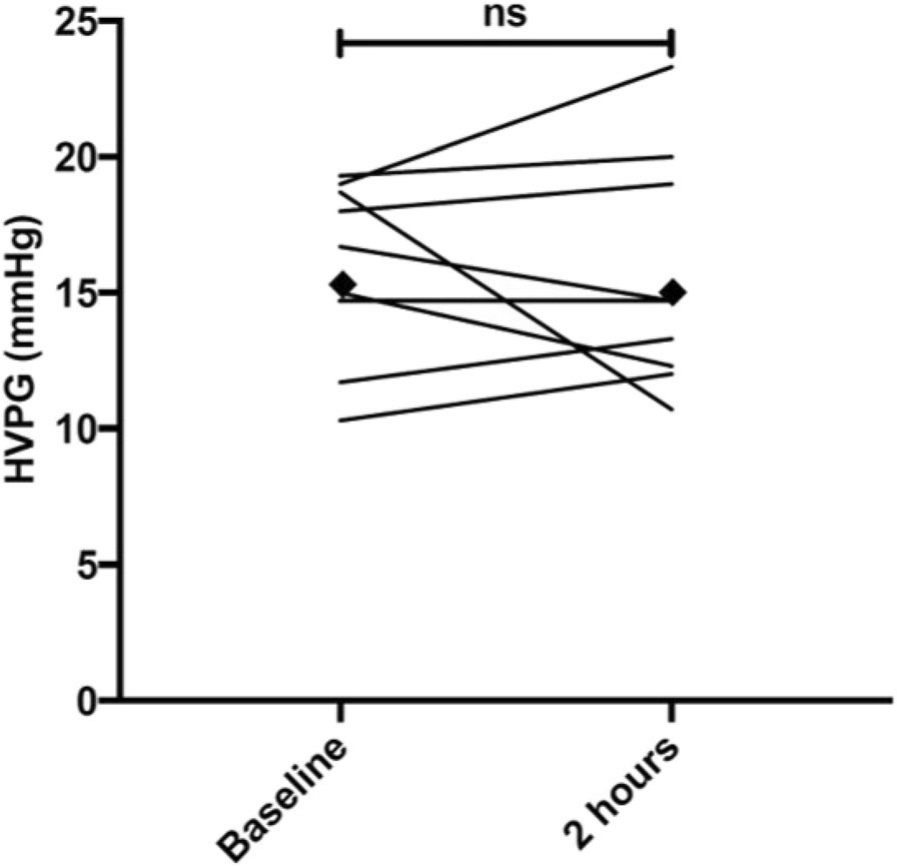
Fasting HVPG response to serelaxin. Lines represent individual participant changes in HVPG following 2-h infusion of serelaxin (*n* = 9). Filled diamonds indicate the group mean at each time point. HVPG hepatic venous pressure gradient, ns not significant

In those allocated to the serelaxin arm (*n* = 11), two participants were withdrawn. The arithmetic mean ± SD HVPG at baseline was 15.9 ± 3.3 mmHg compared to 15.6 ± 4.3 mmHg after 2 h of serelaxin infusion. Although individual responses were variable (Fig. 2), there was no evidence of a significant change in the fasting HVPG between the baseline and 2 h time point (arithmetic mean of difference ± SD was 0.4 ± 3.5 mmHg (95% CI –2.3, 3.1; *p* = 0.76). In the two placebo-treated patients, the HVPG decreased over the 2-h observation period, but no further analysis or conclusions can be made in such a small sample (Table 2).

**Table 2:** Primary and secondary endpoints in participants receiving serelaxin

	Change from baseline to 1 h	Change from baseline to 2 h					
	HVPG (mmHg)	HVPG (mmHg)	HBF (ml/min)	IVCP (mmHg)	Cardiac index (L/min/m^2^)	SVRI (dyne·s·cm^5^·m^2^)	APWV (m/s)
Mean ± SD pre serelaxin	15.9 ± 3.3	15.9 ± 3.3	1.5 ± 0.8	8.2 ± 3.4	3.8 ± 0.5	1716 ± 398	8.1 ± 1.4
Mean ± SD post serelaxin	15.9 ± 2.1	15.6 ± 4.3	1.2 ± 0.8	9.0 ± 2.4	4.1 ± 0.8	1605 ± 474	8.0 ± 2.1
Arithmetic mean of difference ± SD (95% CI)	−0.4 ± 1.9 (−3.4, 2.6), *p* = 0.69	0.4 ± 3.5 (−2.3, 3.1), *p* = 0.76	−0.3 ± 0.3 (− 0.7, 0.1), *p* = 0.15	0.4 ± 1.7 (1.7, −1.2), *p* = 0.58	−0.3 ± 0.7 (− 0.8, 0.3), *p* = 0.28	111 ± 394 (− 192, 414), *p* = 0.42	0.2 ± 0.7 (− 0.4, 0.8), *p* = 0.49
Geometric mean of difference + CV (95% CI)	1.0 + 0.1 (0.8, 1.2), *p* = 0.63	1.0 + 0.2 (0.9, 1.2), *p* = 0.68	0.8 + 0.2 (0.6, 1.1), *p* = 0.15	1.0 + 0.1 (1.0, 1.1), *p* = 0.27	1.0 + 0.1 (0.9, 1.1), *p* = 0.44	1.0 + 0.0 (1.0, 1.0), *p* = 0.32	1.0 + 0.0 (1.0, 1.1), *p* = 0.19

Descriptive statistics for the change from baseline in the HVPG and other endpoints, and the results from paired-sample *t* tests using both the arithmetic mean and the geometric mean

*APWV* aortic pulse wave velocity, *CI* confidence interval, *CV* coefficient of variation, *HBF* hepatic blood flow, *HVPG* hepatic venous pressure gradient, *IVCP* inferior vena cava pressure, *SD* standard deviation, *SVRI* systemic venous resistance index

### Secondary endpoints

There was no evidence of a significant change in fasting HVPG between baseline (15.6 ± 3.3 mmHg) and 1 h of serelaxin infusion (15.8 ± 2.1 mmHg; *p* = 0.63). However, five participants did not have HVPG measurements taken at the 1-h time point. This was due to a decision by the study team to focus efforts on maintaining the catheter position for the critical 2-h HVPG measurement (primary endpoint).

For all of the other secondary endpoints, which were measured after 2 h of serelaxin infusion (including HBF, IVCP, CI, SVRI and APWV), no substantial changes were observed (data summarised in Table 2).

### Safety and tolerability of serelaxin

Treatment with serelaxin was well-tolerated. Overall, 12 AEs were reported in 7 participants treated with serelaxin (Table 3). None were serious or considered related to the IMP. There were no serious adverse events (SAEs) in this study. No pregnancies were reported. There were no striking changes in laboratory blood tests monitored during the course of the study (Table S1 in Additional file 2); in particular, there were no newly occurring liver enzyme abnormalities observed. According to the product label, hypotension is a potential side effect of serelaxin. Patients with cirrhosis and PH often have lower baseline blood pressure levels, predominantly due to severe splanchnic vasodilatation. Following serelaxin, we observed a statistically significant increase in the heart rate (baseline 65 ± 8 bpm vs 2-h post serelaxin 72 ± 8 bpm, *p* = 0.02) and decrease in the mean arterial pressure (baseline 93 ± 7 mmHg vs 2-h post serelaxin 88 ± 5, *p* = 0.02) due to a reduction in diastolic rather than systolic blood pressure (Table S2 in Additional file 2). However, changes were not clinically significant and there were no discontinuations due to tachycardia or hypotension.

**Table 3:** Adverse events

SN	IMP	Adverse event	SAE/SAR	Severity	Relatedness to IMP	Expectedness
002	Placebo	Diarrhoea	No	Mild	N/A	N/A
003	Serelaxin	Syncope on inserting Venflon	No	Mild	Unrelated	Unexpected
003	Serelaxin	Syncope on inserting hepatic venous catheter	No	Mild	Unrelated	Unexpected
003	Serelaxin	Syncope on removing hepatic venous catheter	No	Mild	Unrelated	Unexpected
003	Serelaxin	Right upper-quadrant ache reported at 24 h follow-up	No	Mild	Unrelated	Unexpected
005	Serelaxin	Mean diastolic BP < 60 mmHg (58.3 mmHg at IMP + 30 min)	No	Mild	Possibly related	Expected
006	Serelaxin	Prolonged QTc on ECG after 2 h of serelaxin infusion	No	Mild	Possibly related	Unexpected
008	Serelaxin	Bilirubin rise	No	Mild	Unrelated	Unexpected
012	Serelaxin	Prolonged QTc on ECG during recovery period (normal throughout infusion)	No	Mild	Possibly related	Unexpected
014	Serelaxin	Syncope on inserting Venflon	No	Mild	Unrelated	Unexpected
014	Serelaxin	Syncope on inserting hepatic venous catheter	No	Mild	Unrelated	Unexpected
014	Serelaxin	Dental abscess	No	Mild	Unrelated	Unexpected
016	Serelaxin	Femoral artery puncture	No	Mild	Unrelated	Unexpected

*BP* blood pressure, *ECG* electrocardiogram, *IMP* investigational medicinal product, *N/A* not applicable, *QTc* corrected QT interval, *SAE* serious adverse event, *SAR* serious adverse reaction, *SN* study participant number

## Discussion

In this study, the vasoactive peptide molecule serelaxin (a recombinant form of human relaxin-2) had a neutral effect on the HVPG and a range of secondary haemodynamic endpoints in a population of patients with CSPH (HVPG > 10 mmHg). It is important to note that the trial was terminated before the recruitment target was met; consequently, although there was no overall reduction in the HVPG observed in the serelaxin-treated sample, low statistical power increases the probability of a type II error. However, a consistent finding in this study and previous studies is the good safety profile of serelaxin in patients with liver cirrhosis. With 2 h of serelaxin infusion, there were no newly occurring liver enzyme abnormalities, no clinically significant changes in blood pressure and no discontinuations due to AEs. Additionally, in a separate study, the pharmacokinetic and safety profiles of serelaxin were not affected in patients with mild, moderate or severe hepatic impairment [12]. In contrast, terlipressin is associated with a high risk of serious (particularly ischaemic) complications [17].

PH is the strongest predictor of decompensation and death in patients with compensated cirrhosis [18] and the major driver for serious complications such as variceal bleeding, ascites and hepatic encephalopathy. At present, non-selective beta-blockers, vasopressin analogues and somatostatin analogues are the mainstay of drug treatment for PH, but these strategies are suboptimal and only target splanchnic hyperaemia. New therapeutic options, particularly drugs that reduce increased intrahepatic vascular resistance in cirrhosis, are needed. In preclinical models, serelaxin decreased portal pressure through an increase in intrahepatic nitric oxide (NO) signalling and a reduction in hepatic stellate cell contractility [10]. In an initial small (*n* = 6), exploratory, non-controlled, open-label, phase 2 study, serelaxin induced a rapid and potentially clinically significant reduction in directly measured portal vein pressure (and portal pressure gradient) in patients with cirrhosis, PH and a TIPSS in situ [11].

But pathophysiological mechanisms of PH differ in patients with mild PH (HVPG > 5 mmHg but < 10 mmHg) compared to those with CSPH (HVPG > 10 mmHg) [19]. In mild PH, the main mechanism driving raised portal pressure is increased intrahepatic vascular resistance, while in those with CSPH/varices, increased portal blood flow plays a substantial role in perpetuating and exacerbating the portal hypertensive state. These pathophysiological differences can influence drug efficacy, depending on the stage of disease and the predominant mechanism of action. For example, patients with mild PH have a significantly lower response to non-selective beta-blockers, which reduce portal inflow, compared to those with CSPH/varices who exhibit a hyperdynamic systemic circulation [20]. It is possible, given its proposed mechanism of action in cirrhosis (decreased intrahepatic vascular resistance secondary to increased NO bioavailability), that serelaxin may have a more pronounced effect on portal pressure in patients with mild PH. We recruited patients with HVPG > 10 mmHg because these individuals are at most risk of decompensation and a decrease in portal pressure in this population would potentially lead to a reduction in clinically meaningful endpoints (e.g. development of varices, variceal bleeding and ascites).

The acute haemodynamic effects of vasoactive drugs (e.g. propranolol, terlipressin, octreotide) on portal pressure have generally been demonstrated within 20 min after i.v. administration [8, 21]. Here, serelaxin was administered over a relatively short time-frame (2 h), at least in part because rapid changes in visceral blood flow had been observed in a previous exploratory study in a similar patient population (ClinicalTrials.gov identifier: NCT01640964;). However, for drugs acting on intrahepatic vascular resistance, previous studies have been much longer (e.g. simvastatin significantly decreased the HVPG after 28 days of oral administration) [22]. So, it is conceivable that potential changes in the HVPG due to a reduction in intrahepatic vascular resistance and/or anti-fibrotic/anti-inflammatory mechanisms were not captured after only a short serelaxin infusion. Whether any portal pressure reducing-effect of serelaxin might be demonstrated after prolonged administration would need to be verified in a longer, adequately designed study, if formulation or half-life issues can be resolved to enable chronic exposure to recombinant human relaxin-2 (or alternative relaxin family peptide receptor 1 (RXFP-1) agonist).

### Limitations of the study

The main limitation is that the study was terminated before the recruitment target was met due to slow enrolment and, ultimately, a global drug supply issue (Novartis stopped manufacturing serelaxin and there was none available with a shelf-life beyond 31 August 2018). Therefore, based on the sample size calculation, the study is underpowered to detect the primary endpoint. The study was double-blind and placebo-controlled, which would have addressed potential sources of bias. A formal dose-ranging study of serelaxin in cirrhosis patients has not yet been undertaken. We used the same infusion regimen that had previously shown encouraging haemodynamic effects [23] and had achieved similar steady-state serum concentrations to that observed in our 72-h rat cirrhosis models [10] and in human heart failure following 48 h of i.v. serelaxin infusion [24]. However, the biological effects of relaxin are known to follow a U-shaped dose–response curve [25] and we do not know whether serelaxin might have induced more pronounced effects on the HVPG or secondary haemodynamic endpoints at higher (or lower) doses. Any future work on relaxin effects in cirrhosis should address dose–response relationships.

## Conclusions

In the first randomised controlled trial of serelaxin in patients with liver cirrhosis, an i.v. infusion of serelaxin for 2 h was safe and well-tolerated but caused no significant reduction in portal pressure in participants with CSPH (HVPG > 10 mmHg).

## Supplementary information

**Additional file 1.** Full trial eligibility criteria

**Additional file 2.** Laboratory test results and heart rate and blood pressure measurements

**Additional file 3.** Study protocol

## Data Availability

The datasets supporting the conclusions of this article are included within the article (and its additional files).

## Abbreviations

BP: Blood pressure
GCP: Good Clinical Practice
HR: Heart rate
AE: Adverse event
APWV: Aortic pulse wave velocity
bpm: Beats per minute
CI: Cardiac index
CSPH: Clinically- significant portal hypertension
ECG: Electrocardiogram
FHVP: Free hepatic venous pressure
HVPG: Hepatic venous pressure gradient
ICG: Indocyanine green
IMP: Investigational medicinal product
IVCP: Inferior vena cava pressure
PH: Portal hypertension
PVP: Portal vein pressure
RXFP-1: Relaxin family peptide receptor 1
SAE: Serious adverse event
SVRI: Systemic vascular resistance index
WHVP: Wedged hepatic venous pressure

## References

[1] Williams R, Aspinall R, Bellis M (2014). Addressing liver disease in the UK: a blueprint for attaining excellence in health care and reducing premature mortality from lifestyle issues of excess consumption of alcohol, obesity, and viral hepatitis. Lancet.

[2] Blachier M, Leleu H, Peck-Radosavljevic M (2013). The burden of liver disease in Europe: a review of available epidemiological data. J Hepatol.

[3] Garcia-Tsao G, Groszmann RJ, Fisher RL (1985). Portal pressure, presence of gastroesophageal varices and variceal bleeding. Hepatology.

[4] Ripoll C, Groszmann RJ, Garcia-Tsao G (2009). Hepatic venous pressure gradient predicts development of hepatocellular carcinoma independently of severity of cirrhosis. J Hepatol.

[5] Garcia-Tsao G, Bosch J (2010). Management of varices and variceal hemorrhage in cirrhosis. N Engl J Med.

[6] Tripathi D, Stanley AJ, Hayes PC (2015). U.K. guidelines on the management of variceal haemorrhage in cirrhotic patients. Gut.

[7] Krag A, Borup T, Moller S (2008). Efficacy and safety of terlipressin in cirrhotic patients with variceal bleeding or hepatorenal syndrome. Adv Ther.

[8] Baik SK, Jeong PH, Ji SW (2005). Acute hemodynamic effects of octreotide and terlipressin in patients with cirrhosis: a randomized comparison. Am J Gastroenterol.

[9] Seo YS, Park SY, Kim MY (2014). Lack of difference among terlipressin, somatostatin, and octreotide in the control of acute gastroesophageal variceal hemorrhage. Hepatology.

[10] Fallowfield JA, Hayden AL, Snowdon VK (2014). Relaxin modulates human and rat hepatic myofibroblast function and ameliorates portal hypertension in vivo. Hepatology.

[11] Lachlan NJ, Masson N, Ireland H (2015). Serelaxin reduced portal pressure gradient and portal vein pressure in patients with cirrhosis and portal hypertension [abstract]. Hepatology.

[12] Kobalava Z, Villevalde S, Kotovskaya Y (2015). Pharmacokinetics of serelaxin in patients with hepatic impairment: a single-dose, open-label, parallel group study. Br J Clin Pharmacol.

[13] Harris PA, Taylor R, Thielke R (2009). Research electronic data capture (REDCap)—a metadata-driven methodology and workflow process for providing translational research informatics support. J Biomed Inform.

[14] Tripathi D, Therapondos G, Lui HF (2002). Haemodynamic effects of acute and chronic administration of low-dose carvedilol, a vasodilating beta-blocker, in patients with cirrhosis and portal hypertension. Aliment Pharmacol Ther.

[15] Winkler K, Larsen JA, Munkner T (1965). Determination of the hepatic blood flow in man by simultaneous use of five test substances measured in two parts of the liver. Scand J Clin Lab Invest.

[16] Cherrick GR, Stein SW, Leevy CM (1960). Indocyanine green: observations on its physical properties, plasma decay, and hepatic extraction. J Clin Invest.

[17] Gifford FJ, Morling JR, Fallowfield JA (2017). Systematic review with meta-analysis: vasoactive drugs for the treatment of hepatorenal syndrome type 1. Aliment Pharmacol Ther.

[18] Ripoll C, Groszmann R, Garcia-Tsao G (2007). Hepatic venous pressure gradient predicts clinical decompensation in patients with compensated cirrhosis. Gastroenterology.

[19] Bosch J, Groszmann RJ, Shah VH (2015). Evolution in the understanding of the pathophysiological basis of portal hypertension: how changes in paradigm are leading to successful new treatments. J Hepatol.

[20] Villanueva C, Albillos A, Genesca J (2016). Development of hyperdynamic circulation and response to beta-blockers in compensated cirrhosis with portal hypertension. Hepatology.

[21] Villanueva C, Aracil C, Colomo A (2009). Acute hemodynamic response to beta-blockers and prediction of long-term outcome in primary prophylaxis of variceal bleeding. Gastroenterology.

[22] Abraldes JG, Albillos A, Banares R (2009). Simvastatin lowers portal pressure in patients with cirrhosis and portal hypertension: a randomized controlled trial. Gastroenterology.

[23] Snowdon VK, Lachlan NJ, Hoy AM (2017). Serelaxin as a potential treatment for renal dysfunction in cirrhosis: preclinical evaluation and results of a randomized phase 2 trial. PLoS Med.

[24] Teerlink JR, Cotter G, Davison BA (2013). Serelaxin, recombinant human relaxin-2, for treatment of acute heart failure (RELAX-AHF): a randomised, placebo-controlled trial. Lancet.

[25] Danielson LA, Conrad KP (2003). Time course and dose response of relaxin-mediated renal vasodilation, hyperfiltration, and changes in plasma osmolality in conscious rats. J Appl Physiol.

