# Supplementary material for: A phase 2 randomised controlled trial of serelaxin to lower portal pressure in cirrhosis (STOPP)
Source: Trials. 2020 Mar 12;21:260. doi: 10.1186/s13063-020-4203-9 (PMC7066808; doi:10.1186/s13063-020-4203-9)
Supplement: Supplementary file 1 — Additional file 1. Full trial eligibility criteria [file 13063_2020_4203_MOESM1_ESM.docx]

**SUPPLEMENTARY INFORMATION**

**Additional file 1**

**Full trial eligibility criteria**

***Inclusion criteria*:**

- Ability to provide written informed consent and to understand and willingness to comply with the requirements of the study
- Clinical/imaging-diagnosed or biopsy-proven liver cirrhosis of any aetiology
- Evidence of PH either on imaging or previous endoscopy (patients with large/grade 3 varices as identified by endoscopy within 6 months of screening had to be in an endoscopic band ligation program at the time of study entry)
- Suspected HVPG ≥10 mmHg at baseline (if the baseline HVPG was subsequently found to be <10mmHg on direct measurement, the participant was withdrawn from the study)

***Exclusion criteria*:**

- Pregnancy or breast-feeding
- Women of child-bearing potential not using highly effective methods of contraception
- Severe liver failure defined by one of the following: prothrombin activity < 40%, bilirubin > 5 mg/dL (85 μmol/L), hepatic encephalopathy > grade I
- Presence of any non-controlled and clinically significant disease that could affect the study outcome or that would place the patient at undue risk
- History of variceal bleed within 1 month prior to visit 1
- Hepatocellular carcinoma or history of malignancy of any organ system (other than localised basal cell carcinoma of the skin) treated or untreated
- Portal vein thrombosis
- Previous surgical shunt or TIPSS
- Current use of beta-blockers or nitrates, or any other drug therapy known to have an influence on portal pressure (diuretics were permitted provided patients had been on a stable dose for at least 30 days)
- History of drug or alcohol abuse within 1 month of enrolment
- Sitting systolic blood pressure <110 mmHg at screening visit or within 10 min prior to starting study drug infusion
- Use of other investigational drugs within 5 half-lives of enrolment, or within 30 days/until the expected pharmacodynamic effect had returned to baseline, whichever was longer
- Significant arrhythmias, which included any of the following: sustained ventricular tachycardia, bradycardia with sustained ventricular rate < 45 beats per min or atrial fibrillation/flutter with sustained ventricular response of > 90 beats per min at rest, or long QT syndrome or QTc > 450 msec (QT correction performed using the Fredericia correction method: QTcF = QT/RR0.33) for males and > 460 msec for females at screening visit 1
- Documented hypersensitivity to i.v. contrast agents and/or iodine
- Severe renal impairment (eGFR < 30 mL/min/1.73 m2)
- Significant left ventricular outflow tract obstructions (e.g., severe valvular aortic stenosis, obstructive cardiomyopathy), severe mitral stenosis, restrictive amyloid cardiomyopathy, acute myocarditis; severe aortic or mitral regurgitation for which surgical or percutaneous intervention was indicated
- Major neurologic event including cerebrovascular events, within 30 days prior to screening
- Clinical evidence of acute coronary syndrome currently or within 30 days prior to enrolment
- History of hypersensitivity to study drug serelaxin or study drug ingredients
- Inability to follow instructions or comply with follow-up procedures
- Pacemaker, cardiac resynchronisation device or implantable cardioverter-defibrillator *in situ*
