# Supplementary material for: A phase 2 randomised controlled trial of serelaxin to lower portal pressure in cirrhosis (STOPP)
Source: Trials. 2020 Mar 12;21:260. doi: 10.1186/s13063-020-4203-9 (PMC7066808; doi:10.1186/s13063-020-4203-9)
Supplement: Supplementary file 2 — Additional file 2. Laboratory test results and heart rate and blood pressure measurements [file 13063_2020_4203_MOESM2_ESM.docx]

**SUPPLEMENTARY INFORMATION**

**Additional file 2**

**Table S1. Laboratory test results pre and post serelaxin**

| **Laboratory test** | **Screening visit** (mean ±SD) | **Post serelaxin** (mean ±SD) | **p-value** |
| --- | --- | --- | --- |
| **Haemoglobin** (g/L) | 130 ±13 | 126 ±15 | 0.02 |
| **Total WCC** (x10^9^/L) | 4 ±1 | 5 ±2 | 0.09 |
| **Platelets** (x10^9^/L) | 97 ±57 | 89 ±48 | 0.15 |
| **Prothrombin Time** (s) | 15 ±2 | 16 ±2 | 0.10 |
| **Creatinine** (μmol/L) | 70 ±11 | 67 ±12 | 0.32 |
| **Total bilirubin** (μmol/L) | 27 ±11 | 36 ±18 | 0.02 |
| **AST** (U/L) | 53 ±27 | 52 ±27 | 0.74 |
| **ALT** (U/L) | 46 ±30 | 40 ±17 | 0.33 |
| **Alk Phos** (U/L) | 112 ±38 | 100 ±31 | 0.007 |
| **GGT** (U/L) | 94 ±49 | 95 ±52 | 0.92 |

WCC: white cell count, AST: aspartate aminotransferase, ALT: alanine aminotransferase, Alk Phos: alkaline phosphatase, GGT: gamma-glutamyl transferase.

**Table S2. Heart rate and blood pressure measurements pre and post serelaxin**

|  | **Baseline**  **mean ±SD** | **1 hour post serelaxin**  **mean ±SD, p-value*** | **2 hours post serelaxin**  **mean ±SD, p-value^†^** |
| --- | --- | --- | --- |
| **Heart rate**  **(bpm)** | 65 ±8 | 70 ±10  p=0.06 | 72 ±8  **p=0.02** |
| **Systolic BP (mmHg)** | 133 ±12 | 130 ±15  p=0.23 | 132 ±12  p=0.60 |
| **Diastolic BP (mmHg)** | 73 ±8 | 67 ±6  p=0.005 | 66 ±6  **p=0.001** |
| **MAP**  **(mmHg)** | 93 ±7 | 72 ±36  p=0.02 | 88 ±5  **p=0.02** |

*Comparison baseline to 1 hour, ^†^comparison baseline to 2 hour. BP: blood pressure, MAP: mean arterial pressure
