# Supplementary material for: A phase 2 randomised controlled trial of serelaxin to lower portal pressure in cirrhosis (STOPP)
Source: Trials. 2020 Mar 12;21:260. doi: 10.1186/s13063-020-4203-9 (PMC7066808; doi:10.1186/s13063-020-4203-9)
Supplement: Supplementary file 3 — Additional file 3. Study protocol [file 13063_2020_4203_MOESM3_ESM.pdf]

Study Protocol

Serelaxin To Lower Portal Pressure in Patients with Cirrhosis and Portal Hypertension (STOPP)

Co-sponsors	University of Edinburgh & NHS Lothian<br>ACCORD<br>The Queen's Medical Research Institute<br>47 Little France Crescent<br>Edinburgh<br>EH16 4TJ
Funder	Novartis
Funding Reference Number	
Chief Investigator	Dr Jonathan Fallowfield
EudraCT Number	2015-004031-12
Sponsor Reference	AC15007
REC Number	16/WS/0070
ISRCTN Number	NCT02669875
Version Number and Date	Version 5.0 11 Jun 2018

COORDINATING CENTRE

**Chief Investigator**<br>Dr Jonathan Fallowfield<br>MRC Centre for Inflammation Research<br>Queens Medical Research Institute,<br>47 Little France Crescent,<br>Edinburgh,<br>EH16 4TJ<br>Tel: 0131 242 6655<br>Fax: 0131 242 6682<br>Email: Jonathan.Fallowfield@ed.ac.uk	**Principal Investigator**<br>Dr Fiona Gifford<br>Research Fellow<br>Royal Infirmary of Edinburgh<br>51 Little France Crescent,<br>Edinburgh,<br>EH16 4SA<br>Tel: 0131 242 1712<br>Email: fiona.gifford@nhslothian.scot.nhs.uk
**Co-sponsor Representative**<br>Dr Fiach O'Mahony<br>Research & Development Management Suite<br>E1.12, Queens Medical Research Institute,<br>47 Little France Crescent,<br>Edinburgh,<br>EH164TJ<br>Tel: 0131 242 6226<br>Fax: 0131 242 9418<br>Email: Fiach.O'Mahony@ed.ac.uk	**Co-sponsor Representative**<br>Susan Shepherd<br>Research & Development Management Suite<br>E1.13, Queens Medical Research Institute,<br>47 Little France Crescent,<br>Edinburgh,<br>EH164TJ<br>Tel: 0131 242 3359<br>Email:susan.shepherd@nhslothian.scot.nhs.uk

**Co-Investigator**<br>Professor Peter Hayes<br><br>Liver Unit<br>Royal Infirmary of Edinburgh<br>49 Little France Crescent<br>Edinburgh<br>EH16 4SB<br><br>Tel: 0131 242 1625<br>Email: P.Hayes@ed.ac.uk	**Trial Statistician**<br>Cat Graham<br><br>Wellcome Trust Clinical Research Facility<br>Western General Hospital, Crewe Road<br>South, Edinburgh, EH4 2XU<br><br>Tel: 0131 537 3350<br><br>Email: C.Graham@ed.ac.uk
**Co-Investigator**<br>Dr Philip Dunne<br><br>Liver Unit<br>Royal Infirmary of Edinburgh<br>49 Little France Crescent<br>Edinburgh<br>EH16 4SB<br><br>Tel: 0131 242 1712<br><br>Email: philipdunne@nhs.net	

CONTENTS

1 INTRODUCTION ... 9
1.1 BACKGROUND ... 9
1.2 RATIONALE FOR STUDY ... 9
2 STUDY OBJECTIVES ... 10
2.1 OBJECTIVES ... 10
2.1.1 Primary Objective ... 10
2.1.2 Secondary Objectives ... 10
2.2 ENDPOINTS ... 10
2.2.1 Primary Endpoint ... 10
2.2.2 Secondary Endpoints ... 10
3 STUDY DESIGN ... 11
4 STUDY POPULATION ... 12
4.1 NUMBER OF PARTICIPANTS ... 12
4.2 INCLUSION CRITERIA ... 12
4.3 EXCLUSION CRITERIA ... 13
4.4 CO-ENROLMENT ... 14
5 PARTICIPANT SELECTION AND ENROLMENT ... 14
5.1 IDENTIFYING PARTICIPANTS ... 14
5.2 CONSENTING PARTICIPANTS ... 15
5.3 SCREENING FOR ELIGIBILITY ... 15
5.4 INELIGIBLE AND NON-RECRUITED PARTICIPANTS ... 15
5.5 RANDOMISATION ... 15
5.5.1 Randomisation Procedures ... 15
5.5.2 Treatment Allocation ... 15
5.5.3 Emergency Unblinding Procedures ... 15
5.5.4 Withdrawal of Study Participants ... 16
6 INVESTIGATIONAL MEDICINAL PRODUCT AND PLACEBO ... 17
6.1 STUDY DRUG ... 17
6.1.1 Study Drug Identification ... 17
6.1.2 Study Drug Manufacturer ... 17
6.1.3 Marketing Authorisation Holder ... 17
6.1.4 Labelling and Packaging ... 17
6.1.5 Storage ... 17
6.1.6 Summary of Product Characteristics or Investigators Brochure ... 17
6.2 PLACEBO ... 18
6.3 DOSING REGIME ... 18
6.4 DOSE CHANGES ... 19
6.5 PARTICIPANT COMPLIANCE ... 19
6.6 OVERDOSE ... 19
6.7 OTHER MEDICATIONS ... 19
6.7.1 Non-Investigational Medicinal Products ... 19
6.7.2 Permitted Medications ... 19
6.7.3 Prohibited Medications ... 20
7 STUDY ASSESSMENTS ... 20

7.1 SAFETY ASSESSMENTS 20
7.2 STUDY ASSESSMENTS 21

8 DATA COLLECTION 24

9 STATISTICS AND DATA ANALYSIS 25
9.1 SAMPLE SIZE CALCULATION 25
9.2 PROPOSED ANALYSES 25

10 ADVERSE EVENTS 25
10.1 DEFINITIONS 26
10.2 IDENTIFYING AEs AND SAEs 26
10.3 RECORDING AEs AND SAEs 26
10.4 ASSESSMENT OF AEs AND SAEs 27
10.4.1 Assessment of Seriousness 27
10.4.2 Assessment of Causality 27
10.4.3 Assessment of Expectedness 27
10.4.4 Assessment of Severity 27
10.5 REPORTING OF SAEs/SARs/SUSARs 28
10.6 REGULATORY REPORTING REQUIREMENTS 28
10.7 FOLLOW UP PROCEDURES 29

11 PREGNANCY 30

12 TRIAL MANAGEMENT AND OVERSIGHT ARRANGEMENTS 30
12.1 TRIAL MANAGEMENT GROUP 30
12.2 TRIAL STEERING COMMITTEE 30
12.3 DATA MONITORING COMMITTEE 30
12.4 INSPECTION OF RECORDS 30
12.5 RISK ASSESSMENT 31
12.6 STUDY MONITORING AND AUDIT 31

13 GOOD CLINICAL PRACTICE 31
13.1 ETHICAL CONDUCT 31
13.2 REGULATORY COMPLIANCE 31
13.3 INVESTIGATOR RESPONSIBILITIES 31
13.3.1 Informed Consent 31
13.3.2 Study Site Staff 32
13.3.3 Data Recording 32
13.3.4 Investigator Documentation 32
13.3.5 GCP Training 32
13.3.6 Confidentiality 32
13.3.7 Data Protection 32

14 STUDY CONDUCT RESPONSIBILITIES 33
14.1 PROTOCOL AMENDMENTS 33
14.2 PROTOCOL VIOLATIONS AND DEVIATIONS 33
14.3 SERIOUS BREACH REQUIREMENTS 33
14.4 STUDY RECORD RETENTION 33
14.5 END OF STUDY 33
14.6 CONTINUATION OF DRUG FOLLOWING THE END OF STUDY 34
14.7 INSURANCE AND INDEMNITY 34

15 REPORTING, PUBLICATIONS AND NOTIFICATION OF RESULTS 34

15.1 AUTHORSHIP POLICY ... 34
15.2 PUBLICATION ... 34
15.3 PEER REVIEW ... 34
16 REFERENCES ... 35

PROTOCOL APPROVAL

Serelaxin To Lower Portal Pressure (STOPP) study

EudraCT number 2015-004031-12

Signatures

**Dr Jonathan Fallowfield**<br>Chief Investigator	Signature	Date
**Cat Graham**<br>Trial Statistician	Signature	Date
**Dr Fiach O’Mahony**<br>Sponsor(s) Representative	Signature	Date
**Dr Fiona Gifford**<br>Principal Investigator	Signature	Date
**Professor Peter Hayes**<br>Co-Investigator	Signature	Date

LIST OF ABBREVIATIONS

ACCORD	Academic and Clinical Central Office for Research & Development - Joint office for University of Edinburgh and NHS Lothian
AE	Adverse Event
APWV	Aortic Pulse Wave velocity
AR	Adverse Reaction
BP	Blood Pressure
CI	Cardiac Index
CRF	Case Report Form
ECG	Electrocardiogram
ECTU	Edinburgh Clinical Trials Unit
FHVP	Free Hepatic Venous Pressure
GCP	Good Clinical Practice
HR	Heart Rate
HVPG	Hepatic Venous Pressure Gradient
ICG	Indocyanine Green
ICH	International Conference on Harmonisation
IMP	Investigational Medicinal Product
ISF	Investigator Site File
IVCP	Inferior Vena Cava Pressure
MAP	Mean Arterial Pressure
PHT	Portal Hypertension
PV	Portal Vein
RAP	Right Arterial Pressure
RBF	Renal Blood Flow
SAE	Serious Adverse Event
SAR	Serious Adverse Reaction
SMA	Superior Mesenteric Artery
SOP	Standard Operating Procedure
SUSAR	Suspected Unexpected Serious Adverse Reaction
SVRI	Systemic Vascular Resistance Index
TMF	Trial Master File
UAR	Unexpected Adverse Reaction
WHVP	Wedged Hepatic Venous Pressure

SUMMARY

Cirrhosis (end stage liver scarring) eventually leads to loss of liver function and portal hypertension - an increase in the blood pressure in the portal vein, which carries the blood from the intestine and spleen to the liver. In cirrhosis, the pressure in the portal vein rises because the resistance in the liver is increased because of scarring (fibrosis). As a result, the pressure in the portal vein rises. As the blood tries to find another way back to the heart, new blood vessels open up. Among these vessels are those that run along the wall under the lining of the upper part of the stomach and the lower end of the oesophagus (gullet). These abnormal veins ('varices') protrude into the gullet and the stomach and can bleed without any warning if the pressure rises above a certain threshold. Treatment of bleeding varices includes an endoscopy (where a small flexible tube is put into the gullet to place elastic bands onto the varices to stop the bleeding) and drugs to reduce the risk of further bleeding. Despite a significant improvement in outcomes over the past 3 decades, as many as 1 in 5 people may die following the first episode of variceal bleeding. Current drug therapy consists of substances that work by constricting the blood vessels in the intestine to reduce blood flow into the varices. However, these drugs are ineffective in a proportion of people and also constrict blood vessels elsewhere in the body including the heart and the limbs which can lead to side effects and potentially serious complications. In this randomised placebo-controlled study (STOPP) we aim to look at the effects of a new drug - serelaxin – on portal hypertension by measuring the blood pressure and blood flow in the liver after an infusion for 2 hours through a drip. Serelaxin is a recombinant form of human relaxin-2, which is a naturally occurring protein (hormone) that has been shown in a number of studies in humans to have beneficial effects on the circulation, particularly in patients with heart failure. We have recently shown in a small study in people with cirrhosis and portal hypertension that serelaxin was safe and well tolerated and reduced pressure in the portal vein during a short infusion. We wish to confirm this finding in a larger number of patients using the 'gold standard' test for measuring the effect of drugs on portal hypertension (hepatic venous pressure gradient, HVPG), and to also include a small placebo treated group to confirm that the effects of serelaxin are real and to eliminate any bias in the study by ensuring that participants and investigators do not know which treatment has been allocated (so called 'double blind' study). A reduction in the HVPG by >20% from baseline has been reported to improve clinical outcomes and will be the primary outcome for the study. We will also measure the effects of serelaxin on liver blood flow, on heart function, and on the general circulation to better understand the way in which it works.

1 INTRODUCTION

1.1 BACKGROUND AND RATIONALE FOR STUDY

Standardised mortality rates for liver disease in the UK have increased 400% since 1970, and in people younger than 65 years have increased by almost 500% (Williams R et al, Lancet 2014). In patients with cirrhosis of the liver, portal hypertension is the main cause of death and of liver transplantation. In Europe alone it is estimated that 29 million patients suffer from chronic liver disease, and that 170,000 die each year from complications of cirrhosis, a number exceeding the mortality due to breast cancer (Blachier M et al, J Hepatol 2013). Patients with a hepatic venous pressure gradient (HVPG) >10 mmHg are at increased risk of hepatic decompensation (Garcia-Tsao G et al, Hepatology 1985) and of hepatocellular carcinoma (Ripoll C et al, J Hepatol 2009). Variceal bleeding occurs when the HVPG is >12 mmHg. A reduction in HVPG to <12 mmHg or by >20% from baseline are reported to improve clinical outcomes and represent targets for haemodynamic response in interventional studies (Garcia-Tsao G & Bosch J, NEJM 2010). Despite a significant improvement in outcomes over the past 30 years, the average 6-week mortality of the first episode of variceal bleeding in most studies is reported to be up to 20% (Tripathi D et al, Gut 2015).

Terlipressin, a synthetic analogue of vasopressin, has an immediate systemic vasoconstrictor action followed by portal haemodynamic effects due to slow conversion to vasopressin. It is the only pharmacological agent used in acute variceal bleeding that has been shown to reduce mortality in placebo-controlled trials (Tripathi D et al, Gut 2015). Terlipressin decreases failure of initial haemostasis by 34%, decreases mortality by 34%, and is considered a first-line treatment for bleeding oesophageal varices, when available. However, off-target effects include peripheral and coronary ischaemia, and adverse events (AEs) occur in 10-20% of patients (Krag A et al, Adv Ther 2008). Terlipressin is not licensed in the USA, where octreotide (a somatostatin analogue) is most commonly used. Octreotide is also thought to act as a mesenteric arterial vasoconstrictor, but in an acute haemodynamic study, octreotide was found to only transiently reduce HVPG and portal venous flow (Baik S et al, Am J Gastroenterol 2005). Nevertheless, octreotide has recently been shown to be as effective as terlipressin in the control of acute variceal bleeding (Seo Y et al, Hepatology 2014).

We have previously shown that serelaxin, a recombinant form of the human peptide hormone relaxin-2, has anti-fibrotic and portal hypotensive effects in cirrhotic rats (Fallowfield JA et al, Hepatology 2014). Moreover, serelaxin reduced portal pressure by decreasing intrahepatic vascular resistance through augmentation of nitric oxide (NO) bioavailability and signaling, thus maintaining or enhancing hepatic blood flow. In a recent small exploratory open-label phase II study (EudraCT no. 201200023626, REC ref 12/SS/0177), Part B showed that serelaxin induced a rapid and potentially clinically significant reduction in portal pressure in patients with cirrhosis, portal hypertension and a TIPSS. Following at least 120 minutes of serelaxin infusion there was a 31.3% (95% CI -66.5, 71.6) reduction in the portal pressure gradient (PPG) compared to baseline. During the infusion there was a progressive reduction in the portal vein pressure (PVP) reaching a decrease of 25.2% (95% CI -12.7, 50.3) from baseline at the 120 minute time point. The reduction in PVP started at 30 minutes and continued through to the 135 minute time point. With serelaxin infusion, there were no newly occurring liver enzyme abnormalities, no clinically significant changes in blood pressure, and no discontinuations due to AEs. Indeed, in a separate study the pharmacokinetic and safety profiles of serelaxin were not affected in patients with mild, moderate or severe hepatic impairment (Kobalava Z et al, Br J Clin Pharmacol 2014).

Variceal bleeding and bacterial infections (that frequently occur in patients with cirrhosis and upper gastrointestinal haemorrhage) can precipitate type-1 hepatorenal syndrome, which carries a very high mortality rate. Emerging data suggest that serelaxin may also have renoprotective properties. The beneficial renal haemodynamic effects of serelaxin (increased renal blood flow (RBF) and reduced filtration fraction) have been shown in patients with chronic heart failure (Voors A et al, Circ Heart Fail 2014) and improvement of renal biomarkers (creatinine and cystatin-C) in patients with acute heart failure (Metra M et al, J Am Coll Cardiol 2013). Furthermore, in the recent phase II study (EudraCT no. 201200023626, REC ref 12/SS/0177), Part A showed using 3D phase contrast magnetic resonance angiography that 120 minutes of serelaxin infusion increased RBF by 65.4% from baseline in patients with

cirrhosis and portal hypertension. Serelaxin decreased blood flow in the portal vein (PV) by 11.9%, increased blood flow in the hepatic artery by 18%, but had no effect on superior mesenteric artery (SMA) flow. In contrast, terlipressin markedly reduced SMA flow (36.9%), PV flow (40%) and total liver blood flow (34.7%). Importantly, there was no clinically significant decrease in blood pressure with serelaxin and no difference between pre and post treatment peripheral plasma NO levels.

The potential therapeutic profile of serelaxin (reduction in portal pressure, preserved or increased hepatic blood flow, renal vasodilation, anti-fibrotic) indicates that it may have important effects in patients with chronic liver disease. This randomized placebo-controlled pilot study will evaluate the effect of serelaxin on HVPG and hepatic blood flow in patients with cirrhosis and portal hypertension.

2 STUDY OBJECTIVES

2.1 OBJECTIVES

2.1.1 Primary Objective

To demonstrate that serelaxin induces a clinically significant acute reduction in portal pressure of at least 20% from baseline in patients with cirrhosis and portal hypertension.

2.1.2 Secondary Objectives

- To determine the effect of serelaxin on hepatic blood flow.
- To determine the effect of serelaxin on systemic haemodynamics.
- To collect safety and tolerability data for serelaxin.

2.2 ENDPOINTS

2.2.1 Primary Endpoint

Change from baseline in fasting hepatic venous pressure gradient (HVPG) after 2 hours serelaxin infusion.

2.2.2 Secondary Endpoints

- Change from baseline in fasting hepatic venous pressure gradient (HVPG) after 1 hour serelaxin infusion.
- Change from baseline in fasting hepatic blood flow after 2 hours serelaxin infusion (measured from the concentration of indocyanine green (ICG) in the hepatic venous blood vs peripheral venous blood using the Fick Principle).
- Change from baseline in inferior vena cava pressure (IVCP) after 2 hours serelaxin infusion.
- Change from baseline in cardiac index (CI) after 2 hours serelaxin infusion.
- Change from baseline in systemic vascular resistance index (SVRI) after 2 hours serelaxin infusion.
- Change from baseline in aortic pulse wave velocity after 2 hours serelaxin infusion.
- Safety and tolerability of serelaxin infusion (as assessed throughout the study by monitoring AEs, clinical laboratory blood tests, heart rate, blood pressure and ECG).
- Change from baseline in blood biomarker measurements after 2 hours serelaxin infusion.

The smaller placebo control arm has been included to allow double-blindness (Reverter E et al, Am J Gastroenterol 2015), to control for ‘drift’ of the HVPG over time, and to help generate valuable information for designing a future larger randomised controlled trial.

3 STUDY DESIGN

Study Type: Interventional

Phase: II

Study Design: Allocation: Randomized

Endpoint Classification: Efficacy Study

Intervention Model: Parallel Assignment

Masking: Double-Blind

Primary Purpose: Treatment

Purpose:

This study will investigate the effects of serelaxin on portal hypertension in patients with liver cirrhosis. We will use HVPG and ICG measurement to evaluate the potential benefits of the drug. A preliminary study (EudraCT no. 2012-000236-26, REC ref 12/SS/0177) demonstrated that serelaxin can lower portal pressure.

Study overview:

Participants will attend the Royal Infirmary of Edinburgh Clinical Research Facility (RIE-CRF) for screening (**visit 1**) for less than 60 min consisting of physical examination, screening blood tests (full blood count, coagulation and biochemistry), ECG, blood pressure measurement, and informed consent.

Randomization will be performed once it is known that the participant has passed screening, prior to the study visit. Blocked randomization will be used to achieve balance between study arms and to reduce the opportunity for bias and confounding. Random sequences of block sizes will be generated by computer to achieve a 3:1 allocation ratio between serelaxin and placebo. The randomization service will be managed by the Edinburgh Clinical Trials Unit (ECTU).

At the study visit (**visit 2**; ≤7 days after the screening visit), eligible participants will have baseline haemodynamic measurements performed (described in detail below) following an overnight fast and the avoidance of caffeine for >8 hours. In the unlikely event that >7 days has elapsed, the patient will be rescreened prior to the study visit. After baseline evaluation and confirmation of HVPG ≥10 mmHg, participants will receive (in a double-blind fashion) either serelaxin or placebo. Both treatments will be prepared to be similar in appearance, colour, and organoleptic properties. The haemodynamic measurements will be repeated at specified timepoints (Fig 1). A peripheral blood sample will be taken at baseline and after 2h, processed, and stored as plasma for biomarker measurements. After the post-treatment assessments, participants will be observed for a recovery period of 4h which will include physical examination, blood pressure, ECG measurement, routine laboratory blood tests and repeat urinary pregnancy test.

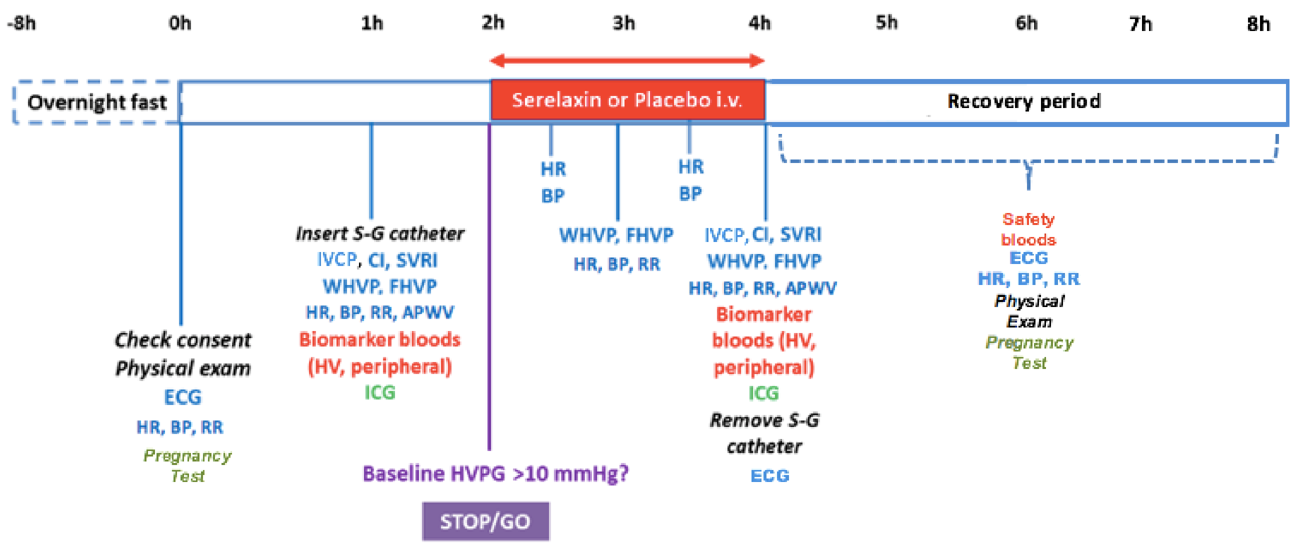

Fig 1. Study visit overview.

ECG, electrocardiogram; HR, heart rate; BP, blood pressure; RR, respiratory rate; S-G, Swan-Ganz; IVCP, inferior vena cava pressure; CI, cardiac index; SVRI, systemic vascular resistance index; APWV, aortic pulse wave velocity; WHVP, wedged hepatic venous pressure; FHVP, free hepatic venous pressure; HVPG, hepatic venous pressure gradient; ICG, indocyanine green; HV, hepatic vein

4 STUDY POPULATION

4.1 NUMBER OF PARTICIPANTS

Planned number of centres: 1

Total number of patients: 20

Timelines and Study duration: 10 months

Start date: 1st November 2016

End date: 30th August 2017

4.2 INCLUSION CRITERIA

1) Male or female adult subjects over 18 years of age

2) Able to provide written informed consent and able to understand and willing to comply with the requirements of the study

3) Clinical/imaging-diagnosed or biopsy-proven liver cirrhosis of any aetiology

4) Evidence of portal hypertension either on imaging or previous endoscopy

5) Patients with large/grade 3 varices as identified by endoscopy within 6 months of screening must be in an endoscopic band ligation programme at the time of study entry

6) Suspected hepatic venous pressure gradient (HVPG) ≥10 mmHg at baseline

4.3 EXCLUSION CRITERIA

1) Pregnancy or nursing (lactating) women
2) Women of child-bearing potential not using highly effective methods of contraception.

- Women of child-bearing potential, defined as all women physiologically capable of becoming pregnant, unless they are using highly effective methods of contraception during the entire period of the study – until 4 weeks following Visit 2.

- Acceptable contraception in women of childbearing age is a "highly effective" contraceptive measure as defined by the Clinical Trials Facilitation Group. This definition includes:

    - Total abstinence (when this is in line with the preferred and usual lifestyle of the subject). Periodic abstinence (e.g., calendar, ovulation, symptothermal, post-ovulation methods) and withdrawal are not acceptable methods of contraception
    - Female sterilization (have had surgical bilateral oophorectomy with or without hysterectomy) total hysterectomy or tubal ligation at least six weeks before taking investigational drug. In case of oophorectomy alone, only when the reproductive status of the woman has been confirmed by follow up hormone level assessment
    - Male sterilization (at least 6 months prior to screening). For female subjects on the study, the vasectomized male partner should be the sole partner for that subject
    - Use of oral, (estrogen and progesterone), injected or implanted hormonal methods of contraception or placement of an intrauterine device (IUD) or intrauterine system (IUS) or other forms of hormonal contraception that have comparable efficacy (failure rate <1%), for example hormone vaginal ring or transdermal hormone contraception

- Further details are found at the following web address: (http://www.hma.eu/fileadmin/dateien/Human_Medicines/01-About_HMA/Working_Groups/CTFG/2014_09_HMA_CTFG_Contraception.pdf

- In case of use of oral contraception women should have been stable on the same pill for a minimum of 3 months before taking the investigational drug.

- Contraception must be continued for up to the end of study – 4 weeks following Visit 2

- Women are considered post-menopausal and not of child-bearing potential if they have had 12 months of natural (spontaneous) amenorrhea with an appropriate clinical profile (e.g. age appropriate, history of vasomotor symptoms) or have had surgical bilateral oophorectomy (with or without hysterectomy), total hysterectomy or tubal ligation at least six weeks ago. In the case of oophorectomy alone, only when the reproductive status of the woman has been confirmed by follow up hormone level assessment is she considered not of child bearing potential.

3) Severe liver failure defined by one of the following: Prothrombin activity < 40%, Bilirubin > 5 mg/dL (85umol/L), hepatic encephalopathy > grade I

4) Presence of any non-controlled and clinically significant disease that could affect the study outcome or that would place the patient at undue risk

5) A history of variceal bleed within 1 month prior to visit 1

6) Hepatocellular carcinoma or history of malignancy of any organ system (other than localized basal cell carcinoma of the skin) treated or untreated.

7) Portal vein thrombosis

8) Previous surgical shunt or TIPSS

9) Current use of beta-blockers or nitrates, any other drug therapy known to have an influence on portal pressure (diuretics permitted provided patients have been on a stable dose for at least 30 days)

10) History of drug or alcohol abuse within 1 month of enrolment

11) Sitting Systolic Blood Pressure <110 mmHg at screening visit or within 10 minutes prior to starting study drug infusion.

12) Use of other investigational drugs within 5 half-lives of enrolment, or within 30 days/until the expected pharmacodynamic effect has returned to baseline, whichever is longer

13) Significant arrhythmias, which include any of the following: sustained ventricular tachycardia, bradycardia with sustained ventricular rate < 45 beats per minute or atrial fibrillation/flutter with sustained ventricular response of > 90 beats per minute at rest, or Long QT syndrome or QTc > 450 msec (QT correction will be performed using the Fridericia correction method: QTcF = QT/RR0.33) for males and > 460 msec for females at screening (visit 1).

14) Documented hypersensitivity to intravenous contrast agents and/or iodine

15) Severe renal impairment (eGFR<30mL/min /1.73m^2)

16) Significant left ventricular outflow tract obstructions (e.g., severe valvular aortic stenosis, obstructive cardiomyopathy), severe mitral stenosis, restrictive amyloid myocardiopathy, acute myocarditis

17) Severe aortic insufficiency or severe mitral regurgitation for which surgical or percutaneous intervention is indicated.

18) Major neurologic event including cerebrovascular events, within 30 days prior to screening

19) Clinical evidence of acute coronary syndrome currently or within 30 days prior to enrolment.

20) History of hypersensitivity to study drug serelaxin or study drug ingredients

21) Inability to follow instructions or comply with follow-up procedures.

22) Permanent pacemaker, cardiac resynchronisation device or implantable cardioverter-defibrillator in situ.

4.4 CO-ENROLMENT

It is possible that participants will have previously been involved in a clinical study. Subjects will not be allowed to enter the study if, at the time of enrolment, they are being treated with any other investigational product or they do not fulfil the Protocol inclusion/exclusion criteria. Participants in the STOPP study cannot enrol in other interventional studies.

5 PARTICIPANT SELECTION AND ENROLMENT

5.1 IDENTIFYING PARTICIPANTS

People with cirrhosis and portal hypertension who are hospital inpatients, or who are attending the endoscopy department or outpatient clinic will be approached initially by a member of the direct care team (e.g. the endoscopist performing the list) if they appear to meet the study eligibility criteria. If an interest is expressed, potential participants will be provided with an information sheet, including contact details of the study investigator.

There will be database searches and review of patient notes in order to identify some potentially eligible patients. Such reviews will be carried out only by employees of the Health Board.

If a member of the research team (i.e. chief/principal/co-investigator) has a treatment relationship with a prospective research participant they may also initially approach that patient about participation.

By invitation letter - potential participants identified by the direct care team may be sent an invitation letter and participant information sheet explaining that there is a study that they may be eligible for. The letter would be sent by a clinical Consultant or Specialist Registrar involved in the patient's care. Should the patient wish to get further information they will be requested to

contact the study centre, at which point the study nurse and/or principal investigator or co-investigator will discuss the study with them. Potentially eligible participants that express interest to participate will be provided with an information sheet for their review as well as having the study explained to them in full by the study nurse, principal investigator or co-investigator.

Potential participants will be given as much time as they need to consider their participation and to discuss this with their family, friends and GP if they wish to.

5.2 CONSENTING PARTICIPANTS

Potentially suitable participants will be given information about the study both verbally (by the principal investigator or co-investigator) and in writing in the form of the patient information sheet. Potential patients will then be given ample time (at least 24 hours) to consider their participation and discuss this with friends, family and/or their GP if required. If participants remain interested, written consent will be taken by the investigator or a suitably qualified and delegated member of the study site staff (i.e. co-investigator) during visit 1 (screening).

5.3 SCREENING FOR ELIGIBILITY

Potential study participants will attend a screening visit at which eligibility (as defined by the inclusion and exclusion criteria) will be assessed. Pre-randomisation screening tests will comprise physical examination (including blood pressure measurement), blood tests and ECG.

Baseline HVPG measurement at the study visit is a stop/go decision point (see Fig 1).

5.4 INELIGIBLE AND NON-RECRUITED PARTICIPANTS

For participants who have given informed consent and are screened but not subsequently randomised, any data/samples already collected may be stored (and used) subject to NHS data management regulations and University of Edinburgh policies intended to safeguard privacy. The standard medical care of ineligible, non-recruited, or non-randomised participants will be unaffected. An anonymised screening log will be maintained and all patients consented for the study will be entered in the screening log. Ineligible and non-recruited persons and the reason for in-eligibility / non-participation will also be documented.

5.5 RANDOMISATION

5.5.1 Randomisation Procedures

Participants will be randomised to receive either serelaxin or placebo. Both treatments will be prepared to be similar in appearance, colour, and organoleptic properties. Masking will be Double-Blind.

Randomization will be carried out after it is confirmed that the participant has passed screening, prior to the study visit (visit 2). The randomization service will be carried out at the Edinburgh Clinical Trials Unit (ECTU), allowing researchers and participants to remain blinded to treatment allocation. Blocked randomisation will be used to achieve balance between study arms and to reduce the opportunity for bias and confounding. Random sequences of block sizes will be generated by computer to achieve a 3:1 allocation ratio between serelaxin and placebo (i.e. n=15:5). Pharmacy will prepare the appropriate treatment after randomisation.

5.5.2 Treatment Allocation

Serelaxin and placebo will be administered, in a double blind manner, via i.v. infusion at two different infusion rates: 80 µg/kg/day for 60 minutes followed by 30 µg/kg/day for at least 60 minutes (until completion of the final HVPG/ICG measurements). This will be achieved by a single infusion bag with a change in the administration rate.

For detailed instructions on preparation and administration of study drug, please refer to the Pharmacy Manual that will be provided separately. All dosages administered to the patient and all dose changes during the study will be recorded.

5.5.3 Emergency Unblinding Procedures

The procedure for emergency unblinding will comply with the European Clinical Trials Directive 2001/20/EC (EUCTD).

The PI is responsible for requesting emergency unblinding. Where the PI delegates emergency unblinding authority to another member of the research team this will be clearly outlined in the clinical trial Delegation Log. A randomisation list will be held by pharmacy.

The study randomisation codes should only be broken for valid medical or safety reasons, for example, a serious adverse event where it is necessary for the investigator or treating healthcare professional to know which treatment the patient is receiving to ensure the participant can receive appropriate urgent safety measures.

If the person requesting the unblinding is the PI they should immediately contact pharmacy during business hours or the on-call pharmacist after hours and request the necessary study codes. If the person requesting the unblinding is not the PI then that healthcare professional should notify the PI (contact information should be stored in the patient medical notes) that unblinding is required for that patient. Where possible it is important that members of the research team remain blinded. Pharmacy should inform the PI or treating healthcare professional with the relevant information as requested. On receipt of the treatment details, the individual providing treatment should attend the participant's medical emergency, as appropriate. If the treating healthcare professional is not the PI, he/she must inform the PI/research team of the code break as soon as possible and the reasons for the actions taken.

The PI is required to document the breaking of the code and the reasons for doing so in the CRF, in the site file, and in the patient's medical notes. Any unblinding must be recorded in the end of study report.

Pharmacy must also document the unblinding and the reasons for doing so on the code list.

The PI is required to notify the Sponsor in writing as soon as possible following the code detailing the reasons for unblinding.

5.5.4 Withdrawal of Study Participants

A participant would be withdrawn from the study if the baseline HVPG measurement is <10 mmHg (as per Fig 1). If upon randomisation it is established that the baseline HVPG is <10 mmHg, the participant would then meet the withdrawal criteria and the study visit would be abandoned.

Participants may voluntarily withdraw from the study for any reason at any time. If premature withdrawal occurs for any reason, the investigator must make every effort to determine the primary reason for a participant's premature withdrawal from the study and record this information on the CRF.

The investigator should discontinue the study treatment for a given participant or withdraw the participant from study if, on balance, he/she believes that continuation would be detrimental to the participant's well-being.

Study treatment must be discontinued under the following circumstances:

- Withdrawal of informed consent
- Emergence of clinically significant adverse events at the discretion of the investigator
- Any other protocol deviation that results in a significant risk to the patient's safety
- Signs or symptoms of hypotension, or blood pressure less than either SBP <90 and/or DBP <60 mmHg should be thoroughly evaluated by the investigator and the patient must be permanently discontinued from study drug.
- If pregnancy is diagnosed at any point during the study.

Patients who are prematurely withdrawn from the study will be replaced by an equal number of newly enrolled patients only if they are discontinued prior to the completion of the final set of haemodynamic measurements (Fig 1).

6 INVESTIGATIONAL MEDICINAL PRODUCT AND PLACEBO

6.1 STUDY DRUG

Serelaxin (recombinant human relaxin-2).

It is a 2-chain heterodimeric molecule, containing a 24 amino acid A chain and a 29 amino acid B chain, covalently bonded by 2 disulfide bridges. The A chain has an additional internal disulfide bridge.

The IMP is being shipped from within the EU:
Roonstrasse, 25
Nuremberg
90429
Germany

6.1.1 Study Drug Identification

Serelaxin (RLX030).

Concentrate For Solution For Infusion (3.5mg/ 3.5mL per vial (1.0mg/mL)).

6.1.2 Study Drug Manufacturer

Novartis Pharma Stein AG

Schaffhauserstrasse

CH-4332 Stein

Switzerland

6.1.3 Marketing Authorisation Holder

The IMP does not have a MA.

6.1.4 Labelling and Packaging

Medication labels will be in the local language and comply with the legal requirements of Annex 13 of the European Union’s Good Manufacturing Practice (GMP). They will include storage conditions for the drug, but no information about the patient.

The formulation is manufactured under GMP using standard aseptic processes, does not contain a preservative and is to be used for single-dose administration only. It is packaged in glass vials with coated rubber stoppers sealed with flip-off caps, which are in common use for packaging of parenteral products.

Study medications will be labelled and packaged in Edinburgh by:

Investigational Supplies Group (ISG)
University of Edinburgh
2nd Floor
1 George Square
Edinburgh, EH8 9JZ, UK
Tel 0131 650 3268

The IMP will be clearly labelled for clinical trial use only.

6.1.5 Storage

Serelaxin (RLX030) will be shipped in bulk, unlabelled, refrigerated (2°C – 8°C [36ºF – 46ºF]), protected from light, and temperature will be monitored during transit (i.e. between manufacturer, ISG and pharmacy).

At site, RLX030 3.5 mg/3.5 mL concentrate for solution for infusion in glass vials will be stored in RIE Pharmacy at 2°C – 8°C (36ºF – 46ºF), protected from light.

Study treatment must be received by a designated person at the study site, handled and stored safely and properly, and kept in a secure location to which only the investigator and designated assistants have access. Upon receipt, all study drugs should be stored according to the instructions specified on the drug labels. Clinical supplies are to be dispensed only in accordance with the protocol.

The investigator must maintain an accurate record of the shipment and dispensing of study drug in a drug accountability ledger. At the conclusion of the study, and as appropriate during the course of the study. The investigator will return all used and unused study drug, packaging, drug labels, and a copy of the completed, anonymised drug accountability ledger to the RIE Pharmacy Department. Any unused study medication will be destroyed by the RIE Pharmacy Department.

6.1.6 Summary of Product Characteristics or Investigators Brochure

Refer to Serelaxin Investigator's Brochure Edition 7.

6.2 PLACEBO

Novartis will supply serelaxin placebo. The placebo used is 20mM sodium acetate buffer solution at pH 5.0 with an appearance identical to serelaxin to achieve blinding.

6.3 DOSING REGIMEN

The dosing regimen for this study is identical to that used in our recent Phase 2 study in cirrhosis and PHT (REC ref: 12/SS/0177) that showed robust effects on renal and hepatic haemodynamics and was safe and well-tolerated.

The pharmacokinetics (PK) of serelaxin has previously been studied in subjects with mild, moderate, and severe hepatic impairment along with healthy control subjects. The study suggests that there is no significant impact of hepatic impairment on the PK of serelaxin (Kobalava et al, 2015). Since the serelaxin infusion time cannot be extended beyond 2 hours for practical reasons (invasive monitoring), modeling and simulation were performed using a population PK approach with the serelaxin PK data from the hepatic impairment study. The goal of the modeling and simulation was to identify a dosing regimen that can achieve serelaxin serum concentrations within less than 2 hours that are comparable to the steady-state concentrations that have shown to be efficacious in patients with acute heart failure in Phase 3 trials. This PK simulation suggests that a dosing with two different infusion rates (80 µg/kg/day for 60 min followed by 30 µg/kg/day for 60 min) would lead to the desired steady-state concentrations within about an hour. This was indeed the case in our previous serelaxin study (REC ref: 12/SS/0177). Furthermore, since serelaxin was expected to be a fast onset treatment, an infusion of 120 min duration was considered sufficient to show pharmacodynamic effects.

The i.v. route of administration was chosen, because it is optimal for the treatment of PHT patients in the acute setting (i.e., acute variceal bleeding). Additionally, it might also render serelaxin less immunogenic than delivery by the s.c. route; no anti-serelaxin antibodies were detected in our previous study in cirrhosis patients.

Table 1 summarises the treatment arms.

TABLE 1: Summary of Investigational and Reference Therapy

Treatment Arm	# of Patients Entered Treatment	Type of Study Drug	Compound	Min Dose	Max Dose	Frequency	Admin. Route	Generic Acceptable? (applies only for comparator)
Arm 1	**15**	Investigational	Serelaxin (RLX030)	30 µg/kg/d	80 µg/kg/d	Continuous infusion 2h	intravenous	-
Arm 2	**5**	Comparator	Placebo	-	-	Continuous infusion 2h	intravenous	No

6.4 DOSE CHANGES

Dose adjustments are not permitted in the study.

6.5 PARTICIPANT COMPLIANCE

Not applicable.

6.6 OVERDOSE

Hypotensive effects would be the major risk following an inadvertent overdose, consistent with the systemic vasodilatation mediated by serelaxin.

In the RELAX-AHF (acute heart failure) study, systolic blood pressure decrease events were more frequent in patients treated with serelaxin (29.4%) than in patients given placebo (18.1%), but generally (84.4% of cases) resolved following either infusion rate adjustment or discontinuation of infusion. Only 12% of these events in the serelaxin group required additional treatment, most being manageable with intravenous fluids, and rarely requiring administration of inotropic agents or mechanical support.

Rescue medication to treat a severe or serious condition in the opinion of the investigator is allowed. Use of rescue medication must be recorded on the CRF.

6.7 OTHER MEDICATIONS

6.7.1 Non-Investigational Medicinal Products

None.

6.7.2 Permitted Medications

Although no formal drug-drug interaction (DDI) studies for serelaxin have been conducted, there is no evidence for apparent mechanisms of potential DDI between serelaxin and other concomitant medications.

In Pre-RELAX-AHF and RELAX-AHF, concomitant medications most commonly used in the standard of care on patients with AHF in the hospital or emergency room setting did not appear to alter the clearance of serelaxin and there was also no clinical indication of adverse events related to DDI.

6.7.3 Prohibited Medications

Use of the treatments listed below is not allowed during the study period. No washout of these prohibited drugs will be performed during or prior to screening with the intention to make a patient eligible for study participation.

Any drug to treat portal hypertension (e.g. vasodilators such as non-selective beta blockers or nitrates). Diuretics are permitted as long as the dose has been stable for the preceding 30 days prior to enrolment.

Action to be taken: Treat as screen failure. Don't administer study treatment / discontinue study treatment. Record on the CRF and screening log.

7 STUDY ASSESSMENTS

7.1 SAFETY ASSESSMENTS

Study-specific safety evaluation will be performed considering changes in systolic and diastolic blood pressure, heart rate, ECG and laboratory tests, as well as physical examination and occurrence of Adverse Events (AEs). All safety parameters will be summarized by treatment group at different time points of the study.

Physical examination

A complete physical examination will be performed at screening (visit 1), and at study visit (visit 2) prior to initiating treatment and in the recovery period after treatment, including the examination of general appearance, heart, lungs, abdomen, height in centimetres (cm) and body weight (to the nearest 0.1 kilogram (kg) in indoor clothing, but without shoes) will be measured.

Information for all physical examinations will be included in the source documentation at the study site. Significant findings that are present prior to the start of study drug will be included in the relevant Past Medical History/Current Medical Conditions screen on the patient's case report form (CRF). Significant findings made after the start of study drug which meet the definition of an AE will be recorded in the AE section of the patient's CRF.

Vital signs

Vital signs include supine blood pressure, heart rate and respiratory rate measurements will be obtained at the specified time points until end of study (Fig 1). Systolic and diastolic blood pressure and heart rate will be measured three times in 1-2 min intervals using an automated validated device, e.g. OMRON, with an appropriately sized cuff. Blood pressure will be measured at baseline (within 10 min of initiating study drug infusion) and every 30 min during the drug treatment and recovery period. The patient's dominant arm will be noted in source documents and used for all measurements while the non-dominant arm will be used for peripheral blood sample collections.

All measurements will be recorded in the patient's source documents and CRF.

ECG

Standard 12 lead ECGs will be performed in triplicate (2±1 min apart) at screening, prior to initiating treatment, at the end of the infusion and in the recovery phase (Fig 1). Interpretation of the tracings will be made by a qualified physician and documented on the ECG section of the CRF. Only clinically significant abnormalities will be reported on this page. Clinically significant abnormalities at screening will also be recorded in the relevant Past Medical History/Current Medical Conditions CRF section. Each ECG tracing will be labelled with the study number, patient initials, patient number, date, and kept in the source documents at the study site.

Laboratory Safety Evaluations

Haematology: Haemoglobin, haematocrit, red blood cell count, white blood cell count with differential, platelet count, and prothrombin time (PT) will be measured at screening visit.

Clinical chemistry: Blood sodium, potassium, urea, creatinine, eGFR, total bilirubin, aspartate aminotransferase (AST), alanine aminotransferase (ALT), alkaline phosphatase, γ-glutamyltransferase (GGT), total protein, and albumin will be measured. If total bilirubin is abnormal, a 'split' bilirubin (direct/indirect bilirubin assay) will be performed.

Pregnancy: All women of child-bearing potential will have a highly sensitive urine pregnancy test performed at screening and at visit 2; both at baseline and at the end of the recovery period. If pregnancy is confirmed the patient will be excluded from study participation.

Appropriateness of Safety Measurements

Since serelaxin was found to be safe in previous studies including the recently completed phase II trial (Protocol no. RLX030X2201, EudraCT no. 201200023626, REC ref. 12/SS/0177) in cirrhosis and portal hypertension, only general measures of safety are to be implemented in the present study (i.e., physical examinations, vital signs, laboratory evaluations (incl. haematology and clinical chemistry), ECG, pregnancy test, collection of AEs and serious adverse events (SAEs)) and no specific additional measures will be implemented.

7.2 STUDY ASSESSMENTS

A summary of study (**visit 2**) assessments is given in Table 3.

Haemodynamic study protocol

All study visit measurements will be performed in the Clinical Research Facility, Royal Infirmary of Edinburgh. All haemodynamic measurements will be performed in the supine position. Low-dose midazolam sedation may be used if required - up to 0.02mg/kg does not alter HVPG measurement (Steinlauf AF et al, Hepatology 1999). After infiltration with up to 10 mL 2% lignocaine (lidocaine), a 7F venous introducer will be inserted into the right femoral vein using the Seldinger technique. Under fluoroscopic guidance, a Swan-Ganz catheter will be inserted through the introducer to measure inferior vena cava pressure, (IVCP, mmHg) in the standard manner. Using the same catheter, the main right hepatic vein will be catheterized for the measurement of the free (FHVP, mmHg) and wedged (WHVP, mmHg) hepatic venous pressures. The HVPG is derived from WHVP - FHVP. All measurements will be performed in triplicate with the mean of the values being used for analysis, and permanent tracings obtained. Permanent tracings will be read blindly at the end of the study before the opening of randomisation codes. The systolic blood pressure (SBP, mmHg) and diastolic blood pressure (DBP, mmHg) will be measured noninvasively using an automatic sphygmomanometer. Aortic pulse wave velocity (m/s), as a measure of arterial function, will be measured noninvasively in the supine position using an inflatable arm cuff (distance pulse wave travels in aorta (suprasternal notch to pubic bone) / measured transit time). Oscillometric recordings will be taken in duplicate. The cardiac output (CO=heart rate x stroke volume/1000, L/min), cardiac index (CI, L/min/m^2) and the systemic vascular resistance index (SVRI, dyne s/cm^5/m^2) will be calculated non-invasively by the bio-impedence technique using the Cardioscreen 1000 Medis (Japp et al, Circulation 2010).
The hepatic blood flow (HBF) will be derived from measurements of ICG clearance and extraction. ICG (ICG-Pulsion, Kimal Plc, 401 Pointon Way, Stonebridge Cross Business Park, Droitwich Spa, Worcestershire, WR9 0LW) will be infused at the beginning as a 10 mg intravenous bolus via a peripheral cannula, followed by an infusion of 0.2 mg/min. After an equilibration time of 40 min, three samples will be taken simultaneously from the right hepatic vein and femoral vein. The HBF will be calculated using the following equation (provided that the hepatic excretion exceeded 10%): HBF = (ICG clearance / ICG extraction) / (1 – haematocrit).

Biomarkers

Peripheral and hepatic venous blood samples will be collected at baseline and after 2h treatment and stored for measurement of blood biomarkers relevant to either the pathophysiology of portal hypertension in cirrhosis or proposed mechanism of drug action.

Analysis of prespecified biomarkers will include: a marker of vasoreactivity (asymmetric dimethylarginine (ADMA)) and a marker of endothelial dysfunction (von Willebrand factor). The list may be changed or expanded further, as more relevant or novel (non-genomic) biomarkers may be discovered during the course of this study.

24 hour and 4 week Follow Up

Participants will be contacted by a member of the research team via telephone 24 hours and again at 4 weeks after Study Visit 2. Participants will be asked open ended questions relating to adverse events and pregnancy. Concomitant medications will also be recorded.

TABLE 3: Summary of Study Assessments

	0	Visit 1	Visit 2				Follow-Up	
	Pre-Screening	Screening	0 hours	1 hour (±15 min)	2 hours (±15 min)	Recovery (4-8 hours)	24 Hours (±3 hours)	4 weeks (±3 days)
Inclusion / Exclusion	X	X	X					
Participant Information Sheet	X							
Informed Consent		X	X[1]					
Demography, Medical History		X	X					
Concomitant Medications		X	X				X	X
Physical Examination		X	X			X		
Height and Weight		X						
Urine Pregnancy Test[2]		X	X			X		
Laboratory Blood Tests		X				X		
Peripheral IV cannula insertions			X					
Femoral Vein cannula insertion			X					
Iodinated contrast (assess catheter position for HVPG)			X	X	X			
HVPG Measurement			X	X	X			
Peripheral Blood Sample (Biomarker Analysis)			X		X			
Hepatic Vein Blood Sample			X		X			
Fluroscopic X-ray Monitoring			X	(X[5])	X			
Thoracic bio-impedance (CI, SVRI)			X		X			
IVCP measurement			X		X			
Aortic Pulse Wave Velocity			X		X			
Indocyanine Green Infusion			X		X			
Blood sampling for Indocyanine Green Measurement			X		X			
Heart Rate Monitoring		X	X	X	X	X		
Respiratory Rate Monitoring		X	X	X	X	X		
BP Monitoring		X	X X[4]	X	X	X		
ECG (in triplicate)		X	X		X	X		
Infusion of Serelaxin / Placebo[3]			X	X	X			
Adverse Events		X	X	X	X	X	X	X
Haemodynamic Measures			X	X	X			
ICG Clearance			X		X			
Biomarkers			X		X			

[1] Ongoing consent will be confirmed at visit 2
[2] Women of childbearing potential only
[3] Infusion of IMP/Placebo will take place as follows: 80 µg/kg/day for 60 minutes followed by 30 µg/kg/day for at least 60 minutes (until completion of the final HVPG/ICG measurements). A single infusion bag will be used with a change in the administration rate at 60 minutes.
[4] Baseline BP measurement must be repeated <10 mins prior to commencing study drug infusion (see exclusion criteria section 4.3).
[5] Optional fluoroscopy, if suspicion that HVPG catheter has moved.

Randomisation will take place the day before Visit 2.

8 DATA COLLECTION

The PI or designated investigator staff will enter the data required by the protocol into the Case Report Form (CRF). A Research Electronic Data Capture (REDCap™) study database will be developed by the ECTU. Participant initials and date of birth will be entered into the database (in line with ECTU SOP), which will be access controlled/password protected, restricting access to the PI and designated investigator staff only. ECTU will maintain the study database server with access/back up as required by regulatory bodies and sponsor (for a period of up to 5 years after database close). In addition, ECTU will provide and maintain documentation relating to testing and validation of the study database in accordance with regulatory and sponsor requirements.

Information to be collected on screening failures

Patients discontinuing prior to receiving study medication are considered screening failures. If a patient has withdrawn before entering the treatment phase, all CRF data, including demographics, vital signs, portal hypertension and cirrhosis history, relevant medical history, and entry with the primary reason for discontinuation should be completed. It is not necessary to complete all the required evaluations unless medically indicated.

Patient demographic and baseline characteristic data

Patient demographic and baseline characteristic data to be collected on all patients include: date of birth, age, sex, race, and ethnicity. Relevant medical history/current medical condition data includes data until the start of study drug. If possible, diagnoses and not symptoms will be recorded. All relevant portal hypertension and cirrhosis history and alcohol status will be reviewed at before administering any study drug.

All medications currently being taken or having been taken up to 30 days prior to entry into the study will be recorded on the Concomitant medications/Significant non-drug therapies section of the CRF. Dosing information for study medication will be collected on the corresponding dosage administration record of the CRF.

Physical examination and vital signs

A complete physical examination will be performed at screening and study completion. It will include the examination of general appearance, heart, lung, and abdomen, thyroid. If indicated, a more detailed exam will be performed. At Screening, height in centimeters (cm) and body weight (to the nearest 0.1 kilogram [kg] in indoor clothing, but without shoes) will be measured. Information for all physical examinations must be included in the source documentation at the study site. Significant findings that are present prior to the start of study drug must be included in the Medical History/Current Medical Conditions screen on the patient's CRF. Significant findings made after the start of study drug which meet the definition of an Adverse Event must be recorded on the Adverse Event screen of the patient's CRF.

Vital signs include sitting blood pressure and pulse measurements and will be obtained at each designated timepoint until end of study. All measurements will be recorded in the CRF and patient's source documents.

Sample Storage and Analysis

Routine blood test samples will be collected in the RIE Clinical Research Facility and analysed locally in NHS laboratory facilities. Laboratory blood results will be entered into the CRF and a paper copy will also be filed in the patient's case notes. Biomarker assays will be undertaken on anonymised blood samples in the MRC Centre for Inflammation Research (Liver Laboratory) by a suitably qualified researcher under the supervision of the CI. With participants' consent, residual blood samples will be stored in an anonymous format on University of Edinburgh premises and may be used in future research as detailed on the patient information sheet and consent form.

ECGs

Standard 12 lead ECGs will be performed in triplicate at screening, pre and post infusion and during the recovery period. Interpretation of the tracings must be made by a qualified physician and documented on the ECG section of the CRF. Each ECG tracing should be labelled with the study number, patient initials, patient number, date, and kept in the source documents at the study site.

Haemodynamic Data Collection

Haemodynamic measurements will be recorded at specified timepoints by the PI or designated investigator staff and entered into the CRF. All HVPG measurements will be performed in triplicate and permanent tracings obtained. Permanent tracings will be kept in the source documents at the study site and read blindly at the end of the study.

9 STATISTICS AND DATA ANALYSIS

9.1 SAMPLE SIZE CALCULATION

The primary efficacy endpoint is the decrease in fasting HVPG between baseline and 2h post serelaxin treatment, targeting for a 20% reduction. The sample size calculation is based on a previous study in Edinburgh evaluating carvedilol (Tripathi D et al, Aliment Pharmacol Ther 2002) and the data from the recent serelaxin phase II study (RLX030X2201). Assuming a mean baseline HVPG of 16.37 (SD=2.14) mmHg and post-baseline HVPG of 13.1 (SD=3.91) mmHg (20% decrease), the change from baseline in HVPG is estimated to be 3.3 (SD=4) mmHg. A sample size of 14 subjects in the serelaxin group will provide 80% power to detect at least a 20% decrease from baseline in HVPG using a two-sided paired t-test with alpha level 0.05. A small number of placebo-treated patients will be included in order to preserve double-blindness, not as a comparison group. Therefore, a total of 20 patients (15 serelaxin and 5 placebo) will be randomized in a 3:1 ratio. For previous studies drawing from a similar patient demographic we have achieved a recruitment rate of 2-3 subjects per month, so the proposed recruitment target is achievable within a 10 month timeframe.

9.2 PROPOSED ANALYSES

Summary statistics (n, mean, SD, median, min, max, Q1 and Q3) will be presented over time for the baseline, post-baseline and change from baseline measurements for the primary endpoint in the serelaxin and placebo group. The geometric mean will be presented for the baseline value, post-baseline values, and for the ratio to the baseline values. Confidence intervals will be calculated for both the arithmetic and geometric means. Paired t-test will be used to test the mean change from baseline measurements. The secondary endpoints (e.g. hepatic blood flow) will be subjected to the same analysis as the HVPG.

The placebo control group will be used to maintain the blind. We will present the baseline to 2 hour change in the same way as the primary outcome although as this has not been powered for, no direct statistical comparison will be made between serelaxin and placebo. The proposed analyses will help gain valuable information for designing a future larger randomised controlled trial.

There is no planned interim data analysis.

Missing data as a result of patients not having the post baseline measurement will not be imputed. Patients with missing post baseline data will be excluded from the analysis at that time point.

10 ADVERSE EVENTS

The Investigator is responsible for the detection and documentation of events meeting the criteria and definitions detailed below.

Full details of contraindications and side effects that have been reported following administration of the IMP can be found in the relevant Investigator's Brochure (IB).

Participants will be instructed to contact their Investigator at any time after consenting to join the trial if any symptoms develop. All adverse events (AE) that occur after signing the consent form must be recorded in detail in the Case Report Form (CRF) or AE form. In the case of an AE, the Investigator should initiate the appropriate treatment according to their medical judgment.

10.1 DEFINITIONS

An **adverse event** (AE) is any untoward medical occurrence in a clinical trial participant which does not necessarily have a causal relationship with an investigational medicinal product (IMP).

An **adverse reaction** (AR) is any untoward and unintended response to an IMP which is related to any dose administered to that participant.

A **serious adverse event** (SAE), **serious adverse reaction** (SAR). Any AE or AR that at any dose:

- results in death of the clinical trial participant;
- is life threatening*;
- requires in-patient hospitalisation^ or prolongation of existing hospitalisation;
- results in persistent or significant disability or incapacity;
- consists of a congenital anomaly or birth defect;
- results in any other significant medical event not meeting the criteria above.

*Life-threatening in the definition of an SAE or SAR refers to an event where the participant was at risk of death at the time of the event. It does not refer to an event which hypothetically might have caused death if it were more severe.

^Any hospitalisation that was planned prior to randomisation will not meet SAE criteria. Any hospitalisation that is planned post randomisation will meet the SAE criteria.

A suspected unexpected serious adverse reaction (SUSAR) is any AR that is classified as serious and is suspected to be caused by the IMP, that it is not consistent with the information about the IMP in the Summary of Product Characteristics (SmPC) or Investigators Brochure.

10.2 IDENTIFYING AEs AND SAEs

All AEs and SAEs will be recorded from the time a participant signs the consent form to take part in the study until **4 weeks after stopping the IMP.** Patients will receive a follow-up phone call four weeks after completion of the study to enquire about AEs and SAEs.

Participants will be asked about the occurrence of AEs/SAEs at every visit during the study. Open-ended and non-leading verbal questioning of the participant will be used to enquire about AE/SAE occurrence. Participants will also be asked if they have been admitted to hospital, had any accidents, used any new medicines or changed concomitant medication regimens. If there is any doubt as to whether a clinical observation is an AE, the event will be recorded.

AEs and SAEs may also be identified via information from support departments e.g. laboratories.

10.3 RECORDING AEs AND SAEs

When an AE/SAE occurs, it is the responsibility of the Investigator or another suitably qualified physician in the research team who is delegated to record and report AEs/SAEs to review all documentation (e.g. hospital notes, laboratory and diagnostic reports) related to the event. The Investigator will then record all relevant information in the CRF and on the SAE form (if the AE meets the criteria of serious).

Information to be collected includes dose, type of event, onset date, Investigator assessment of severity and causality, date of resolution as well as treatment required, investigations needed and outcome.

10.4 ADVERSE EVENT REPORTING FOR THIS TRIAL

All adverse events for each participant will be recorded on the AE log and will be assigned the appropriate MedDRA Systems Organ Class (SOC) code. Upon completion of the trial, the Adverse Event Log for that participant will be transmitted electronically to ACCORD. This must be received by ACCORD within 6 weeks of the final participant completing the trial.

The de-identified Adverse Event Log will be transmitted via email to safety@accord.scot or by fax to ACCORD on +44 (0)131 242 9447 or may be transmitted by hand to the office.

10.4.1 PRE-EXISTING MEDICAL CONDITIONS

Pre-existing medical conditions (i.e. existed prior to informed consent) should be recorded as medical history and only recorded as adverse events if medically judged to have worsened during the study.

10.4.2 WORSENING OF THE UNDERLYING CONDITION DURING THE TRIAL

Medical occurrences or symptoms of deterioration in the participant's underlying condition will be recorded as AEs.

10.5 ASSESSMENT OF AEs AND SAEs

Each AE must be assessed for seriousness, causality, severity and ARs must be assessed for expectedness by the Principal Investigator or another suitably qualified physician in the research team who has been delegated this role.

For randomised double blind studies, AEs will be assessed as though the participant is taking active IMP. SUSARs will be unblinded by ACCORD before they are reported to REC and CA (by ACCORD).

The Chief Investigator (CI) may not downgrade an event that has been assessed by an Investigator as an SAE or SUSAR, but can upgrade an AE to an SAE, SAR or SUSAR if appropriate.

10.5.1 Assessment of Seriousness

The Investigator will make an assessment of seriousness as defined in Section 10.1.

10.5.2 Assessment of Causality

The Investigator will make an assessment of whether the AE/SAE is likely to be related to the IMP according to the definitions below.

- Unrelated: where an event is not considered to be related to the IMP.

- Possibly Related: The nature of the event, the underlying medical condition, concomitant medication or temporal relationship make it possible that the AE has a causal relationship to the study drug. The assessment of causality will be made against the reference safety information found in the Serelaxin Investigator's Brochure Edition 5 (section 5.2).

Where non Investigational Medicinal Products (NIMPs) e.g. rescue/escape drugs are given: if the AE is considered to be related to an interaction between the IMP and the NIMP, or where the AE might be linked to either the IMP or the NIMP but cannot be clearly attributed to either one of these, the event will be considered as an AR. Alternative causes such as natural history of the underlying disease, other risk factors and the temporal relationship of the event to the treatment should be considered and investigated. The blind should not be broken for the purpose of making this assessment.

10.5.3 Assessment of Expectedness

If an event is judged to be an AE, the evaluation of AE expectedness will be made based on the Reference Safety Information of the IB in effect. The IB contains a list of AE(s) considered to be 'expected for reporting purposes'. In accordance with this list, the AE will be determined as expected or unexpected.

10.5.4 Assessment of Severity

The Investigator will make an assessment of severity for each AE/SAE and record this on the CRF or SAE form according to one of the following categories:

Mild: an event that is easily tolerated by the participant, causing minimal discomfort and not interfering with every day activities.

Moderate: an event that is sufficiently discomforting to interfere with normal everyday activities.

Severe: an event that prevents normal everyday activities.

Note: the term 'severe', used to describe the intensity, should not be confused with 'serious' which is a regulatory definition based on participant/event outcome or action criteria. For example, a headache may be severe but not serious, while a minor stroke is serious but may not be severe.

10.6 REPORTING OF SAEs/SARs/SUSARs

Once the Investigator becomes aware that an SAE has occurred in a study participant, the information will be reported to the ACCORD Research Governance & QA Office **immediately or within 24 hours**. If the Investigator does not have all information regarding an SAE, they should not wait for this additional information before notifying ACCORD. The SAE report form can be updated when the additional information is received.

The SAE report will provide an assessment of causality and expectedness at the time of the initial report to ACCORD according to Sections 10.4.2, Assessment of Causality and 10.4.3, Assessment of Expectedness.

The SAE form will be transmitted by fax to ACCORD on **+44 (0)131 242 9447** or may be transmitted by hand to the office or submitted via email to safety@accord.scot. Only forms in a pdf format will be accepted by ACCORD via email.

Where missing information has not been sent to ACCORD after an initial report, ACCORD will contact the investigator and request the missing information. The Investigator must respond to these requests in a timely manner.

All reports faxed to ACCORD and any follow up information will be retained by the Investigator in the Investigator Site File (ISF).

Required information will be transferred to Novartis within the required timelines shown below in Table 4.

Table 4

Information collected by Sponsor	Information to be transferred to Novartis within 15 days of awareness of event

• All SAEs<br>• All reports of drug exposure during pregnancy<br>• All AEs<br>• All reports of misuse and abuse of an IMP, other medication errors and uses outside of what is foreseen in the protocol (irrespective if a clinical event has occurred)	• All collected SAEs in subjects exposed to the Novartis IMP**<br>• All collected pregnancy reports in subjects exposed to the Novartis IMP**<br>• All collected reports of abuse and misuse of the Novartis IMP**<br><br>-Individual transfer of non-serious AEs is not required (unless specifically required for that particular protocol)<br>- Individual transfer of reports of medication errors and uses outside of what is foreseen in the protocol is not required (unless these are associated with an SAE)<br><br>**For blinded studies, this also includes reports to the blinded medication

10.7 REGULATORY REPORTING REQUIREMENTS

The ACCORD Research Governance & QA Office is responsible for pharmacovigilance reporting on behalf of the co-sponsors (Edinburgh University and NHS Lothian).

The ACCORD Research Governance & QA Office has a legal responsibility to notify the regulatory competent authority and relevant ethics committee (Research Ethics Committee (REC) that approved the trial). Fatal or life threatening SUSARs will be reported no later than 7 calendar days and all other SUSARs will be reported no later than 15 calendar days after ACCORD is first aware of the reaction.

ACCORD will inform Investigators at participating sites of all SUSARs and any other arising safety information.

ACCORD will be responsible for providing safety line listings and assistance; however, it is the responsibility of the Investigator to prepare the Development Safety Update Report. This annual report lists all SARs and SUSARs reported during that time period. The responsibility of submitting the Development Safety Update Report to the regulatory authority and RECs, lies with ACCORD.

10.8 FOLLOW UP PROCEDURES

After initially recording an AE or recording and reporting an SAE, the Investigator should make every effort to follow each event until a final outcome can be recorded or reported as necessary. Follow up information on an SAE will be reported to the ACCORD office.

If, after follow up, resolution of an event cannot be established, an explanation should be recorded on the CRF or AE log or additional information section of SAE form

11 PREGNANCY

Pregnancy is not considered an AE or SAE; however, to ensure patient safety, the Investigator will collect pregnancy information for any female participants or female partners of male participants who become pregnant while participating in the study. The Investigator will record the information on a Pregnancy Notification Form and submit this to the ACCORD office within 24 hours of being made aware of the pregnancy.

The pregnancy should be followed up to determine outcome, including spontaneous or voluntary termination, details of the birth, and the presence or absence of any birth defects, congenital abnormalities, or maternal and/or newborn complications.

Any SAE experienced during the pregnancy and unrelated to the pregnancy must be reported on a SAE form.

If the investigator becomes aware that the female partner of a male participant has become pregnant then consent should be sought from the female partner to collect pregnancy outcomes. Additionally, consent to report information regarding these pregnancy outcomes should be obtained from the female partner.

12 TRIAL MANAGEMENT AND OVERSIGHT ARRANGEMENTS

12.1 TRIAL MANAGEMENT GROUP

The trial will be coordinated by a Project Management Group consisting of the grant holders (Chief Investigator and co-Investigators in Edinburgh), coordinating nurse(s) and Data Manager.

The Chief Investigator will oversee the study. The Data Manager in ECTU will be responsible for checking the CRFs for completeness, plausibility and consistency. Any queries will be resolved by the Investigator or delegated member of the trial team.

The ECTU office will be responsible for randomization, collection of data in collaboration with the investigators/research nurses and data processing.

A Delegation Log will be prepared for each site, detailing the responsibilities of each member of staff working on the trial.

12.2 TRIAL STEERING COMMITTEE

No formal Trial Steering Committee (TSC) will be established as this is a small, single centre study.

12.3 DATA MONITORING COMMITTEE

An independent Data Monitoring Committee (DMC) will not be established to oversee the safety of participants in the trial. This short-term study follows on from a recently completed phase 2 study at the same site, in a similar population of adult patients with portal hypertension, with the same primary endpoint, where serelaxin was found to be safe, well tolerated and there were no discontinuations due to AEs.

12.4 INSPECTION OF RECORDS

Investigators and institutions involved in the study will permit trial related monitoring and audits on behalf of the sponsor, REC review, and regulatory inspection(s). In the event of an audit or monitoring, the Investigator agrees to allow the representatives of the sponsor direct access to all study records and source documentation. In the event of regulatory inspection, the Investigator agrees to allow inspectors direct access to all study records and source documentation.

12.5 RISK ASSESSMENT

A study specific risk assessment will be performed by representatives of the co-sponsors, ACCORD monitors and the QA group, in accordance with ACCORD governance and sponsorship SOPs. Input will be sought from the Chief Investigator or designee. The outcomes

of the risk assessment will form the basis of the monitoring plans and audit plans. The risk assessment outcomes will also indicate which risk adaptions (delete if no adaptations were possible) could be incorporated into to trial design.

12.6 STUDY MONITORING AND AUDIT

ACCORD clinical trial monitors, or designees, will perform monitoring activities in accordance with the study monitoring plan. This will involve on-site visits and central monitoring activities as necessary (delete where not required). ACCORD QA personnel, or designees, will perform study audits in accordance with the study audit plan. This will involve investigator site audits, study management audits and facility (including 3rd parties) audits as necessary (delete where not required).

13 GOOD CLINICAL PRACTICE

13.1 ETHICAL CONDUCT

The study will be conducted in accordance with the principles of the International Conference on Harmonisation Tripartite Guideline for Good Clinical Practice (ICH GCP).

A favorable ethical opinion will be obtained from the appropriate REC and local R&D approval will be obtained prior to commencement of the study.

13.2 REGULATORY COMPLIANCE

The study will not commence until a Clinical Trial Authorisation (CTA) is obtained from the appropriate Regulatory Authority. The protocol and study conduct will comply with the Medicines for Human Use (Clinical Trials) Regulations 2004, as amended.

13.3 INVESTIGATOR RESPONSIBILITIES

The Investigator is responsible for the overall conduct of the study at the site and compliance with the protocol and any protocol amendments. In accordance with the principles of ICH GCP, the following areas listed in this section are also the responsibility of the Investigator. Responsibilities may be delegated to an appropriate member of study site staff.

13.3.1 Informed Consent

The Investigator is responsible for ensuring informed consent is obtained before any protocol specific procedures are carried out. The decision of a participant to participate in clinical research is voluntary and should be based on a clear understanding of what is involved.

Participants must receive adequate oral and written information – appropriate Participant Information and Informed Consent Forms will be provided. The oral explanation to the participant will be performed by the Investigator or qualified delegated person, and must cover all the elements specified in the Participant Information Sheet and Consent Form.

The participant must be given every opportunity to clarify any points they do not understand and, if necessary, ask for more information. The participant must be given sufficient time to consider the information provided. It should be emphasised that the participant may withdraw their consent to participate at any time without loss of benefits to which they otherwise would be entitled.

The participant will be informed and agree to their medical records being inspected by regulatory authorities and representatives of the sponsor(s) but understand that their name will not be disclosed outside the hospital.

The Investigator or delegated member of the trial team and the participant will sign and date the Informed Consent Form(s) to confirm that consent has been obtained. The participant will receive a copy of this document and a copy filed in the Investigator Site File (ISF) and participant's medical notes.

13.3.2 Study Site Staff

The Investigator must be familiar with the IMP, protocol and the study requirements. It is the Investigator's responsibility to ensure that all staff assisting with the study are adequately informed about the IMP, protocol and their trial related duties.

13.3.3 Data Recording

The Principal Investigator is responsible for the quality of the data recorded in the CRF at each Investigator Site. The source data plan identifies which source data correspond to CRF data and states which data are recorded directly into the CRF. The ECTU Data Manager will set up basic data queries requested by the Investigator as capability of REDCap will allow.

13.3.4 Investigator Documentation

Prior to beginning the study, each Investigator will be asked to provide particular essential documents to the ACCORD Research Governance & QA Office, including but not limited to:

- An original signed Investigator's Declaration (as part of the Clinical Trial Agreement documents);
- Curriculum vitae (CV) signed and dated by the Investigator indicating that it is accurate and current.

The ACCORD Research Governance & QA Office will ensure all other documents required by ICH GCP are retained in a Trial Master File (TMF), where required, and that appropriate documentation is available in local ISFs.

13.3.5 GCP Training

All study staff must hold evidence of appropriate GCP training.

13.3.6 Confidentiality

All laboratory specimens, evaluation forms, reports, and other records must be identified in a manner designed to maintain participant confidentiality. All records must be kept in a secure storage area with limited access. Clinical information will not be released without the written permission of the participant. The Investigator and study site staff involved with this study may not disclose or use for any purpose other than performance of the study, any data, record, or other unpublished, confidential information disclosed to those individuals for the purpose of the study. Prior written agreement from the sponsor or its designee must be obtained for the disclosure of any said confidential information to other parties.

13.3.7 Data Protection

All Investigators and study site staff involved with this study must comply with the requirements of the Data Protection Act 1998 with regard to the collection, storage, processing and disclosure of personal information and will uphold the Act's core principles. Access to collated participant data will be restricted to those clinicians treating the participants, representatives of the sponsor(s) and representatives of regulatory authorities.

Computers used to collate the data will have limited access measures via user names and passwords.

Published results will not contain any personal data that could allow identification of individual participants.

14 STUDY CONDUCT RESPONSIBILITIES

14.1 PROTOCOL AMENDMENTS

Any changes in research activity, except those necessary to remove an apparent, immediate hazard to the participant in the case of an urgent safety measure, must be reviewed and approved by the Chief Investigator.

Amendments to the protocol must be submitted in writing to the appropriate REC, Regulatory Authority and local R&D for approval prior to participants being enrolled into an amended protocol.

14.2 PROTOCOL VIOLATIONS AND DEVIATIONS

Prospective protocol deviations, i.e. protocol waivers, will not be approved by the sponsors and therefore will not be implemented, except where necessary to eliminate an immediate hazard to study participants. If this necessitates a subsequent protocol amendment, this should be submitted to the REC, Regulatory Authority and local R&D for review and approval if appropriate.

Protocol deviations will be recorded in a protocol deviation log and logs will be submitted to the sponsors every 3 months. Each protocol violation will be reported to the sponsor within 3 days of becoming aware of the violation.

14.3 SERIOUS BREACH REQUIREMENTS

A serious breach is a breach which is likely to effect to a significant degree:

(a) the safety or physical or mental integrity of the participants of the trial; or

(b) the scientific value of the trial.

If a potential serious breach is identified by the Chief investigator, Principal Investigator or delegates, the co-sponsors (accord.seriousbreach@ed.ac.uk) must be notified within 24 hours. It is the responsibility of the co-sponsors to assess the impact of the breach on the scientific value of the trial, to determine whether the incident constitutes a serious breach and report to regulatory authorities and research ethics committees as necessary.

14.4 STUDY RECORD RETENTION

The study data will be retained until at least 2 years after the last approval of the marketing application in an ICH region, and until there are no pending contemplated marketing authorisations in an ICH region or at least 2 years have elapsed since the formal discontinuation of clinical development of the investigational product. This equates to approximately 15 years. When the retention period has elapsed, study documentation will not be destroyed without permission from the sponsor.

14.5 END OF STUDY

The end of study is defined as the end of the 4 week follow up period of the final participant.

The Investigators and/or the trial steering committee and/or the co-sponsor(s) have the right at any time to terminate the study for clinical or administrative reasons.

The end of the study will be reported to the REC and Regulatory Authority within 90 days, or 15 days if the study is terminated prematurely. The Investigators will inform participants of the premature study closure and ensure that the appropriate follow up is arranged for all participants involved. End of study notification will be reported to the co-sponsors via email to resgov@accord.scot.

In accordance with ACCORD SOP CR011, a Clinical Study Report (CSR) will be provided to the Sponsor (QA@accord.scot), and REC within 1 year of the end of the study.

Upon completion of the study, the Investigator will upload clinical trial results onto the EudraCT database on behalf of the Sponsor.

The Investigator will submit a short confirmatory e-mail to the MHRA (CT.Submission@mhra.gsi.gov.uk) once the result-related information has been uploaded to EudraCT, with 'End of trial: result-related information: EudraCT XXXX-XXXXXX-XX' as the subject line. The Sponsor(s) will be copied in this e-mail (QA@accord.scot). It should be noted that you will not get an acknowledgment e-mail or letter from the MHRA.

14.6 CONTINUATION OF DRUG FOLLOWING THE END OF STUDY

The IMP will not be continued following the end of the study.

14.7 INSURANCE AND INDEMNITY

The co-sponsors are responsible for ensuring proper provision has been made for insurance or indemnity to cover their liability and the liability of the Chief Investigator and staff.

The following arrangements are in place to fulfil the co-sponsors' responsibilities:

- The Protocol has been designed by the Chief Investigator and researchers employed by the University and collaborators. The University has insurance in place (which includes no-fault compensation) for negligent harm caused by poor protocol design by the Chief Investigator and researchers employed by the University.
- Sites participating in the study will be liable for clinical negligence and other negligent harm to individuals taking part in the study and covered by the duty of care owed to them by the sites concerned. The co-sponsors require individual sites participating in the study to arrange for their own insurance or indemnity in respect of these liabilities.
- Sites which are part of the United Kingdom's Nation Health Service will have the benefit of NHS Indemnity.
- Sites out with the United Kingdom will be responsible for arranging their own indemnity or insurance for their participation in the study, as well as for compliance with local law applicable to their participation in the study.
- The manufacturer supplying IMP has accepted limited liability related to the manufacturing and original packaging of the study drug and to the losses, damages, claims or liabilities incurred by study participants based on known or unknown Adverse Events which arise out of the manufacturing and original packaging of the study drug, but not where there is any modification to the study drug (including without limitation re-packaging and blinding).

15 REPORTING, PUBLICATIONS AND NOTIFICATION OF RESULTS

15.1 AUTHORSHIP POLICY

Ownership of the data arising from this study resides with the study team. On completion of the study, the study data will be analysed and tabulated, and a clinical study report will be prepared in accordance with ICH guidelines.

15.2 PUBLICATION

The clinical study report will be used for publication and presentation at scientific meetings. Investigators have the right to publish orally or in writing the results of the study.

Summaries of results will also be made available to Investigators for dissemination within their clinics (where appropriate and according to their discretion).

15.3 PEER REVIEW

The study has been subjected to independent peer-review by an external expert assessor.

16 REFERENCES

Garcia-Tsao G, Groszmann RJ, Fisher RL, et al. Portal pressure, presence of gastroesophageal varices and variceal bleeding. Hepatology 1985;5: 419–24.

Ripoll C, Groszmann RJ, Garcia-Tsao G et al. Portal Hypertension Collaborative Group. Hepatic venous pressure gradient predicts development of hepatocellular carcinoma independently of severity of cirrhosis. J. Hepatol 2009;50(5):923–928.

Garcia-Tsao G, Bosch J. Management of varices and variceal hemorrhage in cirrhosis. N Engl J Med 2010;362(9):823–832.

Tripathi D, Stanley AJ, Hayes PC et al. UK guidelines on the management of variceal haemorrhage in cirrhotic patients. Gut 2015 doi: 10.1136/gutjnl-2015-309262. [Epub ahead of print]

Krag A, Borup T, Møller S, Bendtsen F. Efficacy and safety of terlipressin in cirrhotic patients with variceal bleeding or hepatorenal syndrome. Adv Ther 2008;25(11):1105-40.

Baik SK, Jeong PH, Ji SW et al. Acute hemodynamic effects of octreotide and terlipressin in patients with cirrhosis: a randomized comparison. Am J Gastroenterol 2005;100(3):631-5.

Seo YS, Park SY, Kim MY, et al. Lack of difference among terlipressin, somatostatin, and octreotide in the control of acute gastroesophageal variceal hemorrhage. Hepatology 2014;60:954–63.

Fallowfield JA, Hayden AL, Snowdon VK et al. Relaxin modulates human and rat hepatic myofibroblast function and ameliorates portal hypertension in vivo. Hepatology 2014;59(4):1492-504

Kobalava Z, Villevalde Y, Kotovskaya H et al. Pharmacokinetics of serelaxin in patients with hepatic impairment: A single-dose, open-label, parallel-group study. Br J Clin Pharmacol 2015;79(6):937-45

Voors A, Dahlke M, Meyer S et al. Renal hemodynamic effects of serelaxin in patients with chronic heart failure: a randomized, placebo-controlled study. Circ Heart Fail 2014;7:994-1002.

Metra M, Cotter G, Davison BA. Effect of serelaxin on cardiac, renal, and hepatic biomarkers in the Relaxin in Acute Heart Failure (RELAX-AHF) development program: correlation with outcomes. J Am Coll Cardiol 2013;61:196-206.

Tripathi D, Therapondos G, Lui HF et al. Haemodynamic effects of acute and chronic administration of low-dose carvedilol, a vasodilating beta-blocker, in patients with cirrhosis and portal hypertension. Aliment Pharmacol Ther 2002;16(3):373-80.

Steinlauf AF, Garcia-Tsao G, Zakko MF, Dickey K, Gupta T, Groszmann RJ. Low-dose midazolam sedation: an option for patients undergoing serial hepatic venous pressure measurements. Hepatology 1999;29(4):1070-3.

Reverter E, Mesonero F, Seijo S et al., Effects of Sapropterin on Portal and Systemic Hemodynamics in Patients With Cirrhosis and Portal Hypertension: A Bicentric Double-Blind Placebo-Controlled Study. Am J Gastroenterol 2015;110(7):985-92.

Japp AG, Cruden NL, Barnes G et al. Acute cardiovascular effects of apelin in humans: potential role in patients with chronic heart failure. Circulation 2010;121(16):1818-27.
